# SARS-CoV-2 Nucleocapsid Protein Has DNA-Melting and Strand-Annealing Activities With Different Properties From SARS-CoV-2 Nsp13

**DOI:** 10.3389/fmicb.2022.851202

**Published:** 2022-07-22

**Authors:** Bo Zhang, Yan Xie, Zhaoling Lan, Dayu Li, Junjie Tian, Qintao Zhang, Hongji Tian, Jiali Yang, Xinnan Zhou, Shuyi Qiu, Keyu Lu, Yang Liu

**Affiliations:** ^1^College of Basic Medicine, Zunyi Medical University, Zunyi, China; ^2^School of Public Health, Zunyi Medical University, Zunyi, China; ^3^Key Laboratory of Plant Resource Conservation and Germplasm Innovation in Mountainous Region (Ministry of Education), College of Life Sciences/Institute of Agro-bioengineering, Guizhou University, Guiyang, China

**Keywords:** SARS-CoV-2, Nsp13, nucleocapsid protein, unwinding, helicase

## Abstract

Since December 2019, severe acute respiratory syndrome coronavirus 2 (SARS-CoV-2) has spread throughout the world and has had a devastating impact on health and economy. The biochemical characterization of SARS-CoV-2 proteins is important for drug design and development. In this study, we discovered that the SARS-CoV-2 nucleocapsid protein can melt double-stranded DNA (dsDNA) in the 5′-3′ direction, similar to SARS-CoV-2 nonstructural protein 13. However, the unwinding activity of SARS-CoV-2 nucleocapsid protein was found to be more than 22 times weaker than that of SARS-CoV-2 nonstructural protein 13, and the melting process was independent of nucleoside triphosphates and Mg^2+^. Interestingly, at low concentrations, the SARS-CoV-2 nucleocapsid protein exhibited a stronger annealing activity than SARS-CoV-2 nonstructural protein 13; however, at high concentrations, it promoted the melting of dsDNA. These findings have deepened our understanding of the SARS-CoV-2 nucleocapsid protein and will help provide novel insights into antiviral drug development.

## Introduction

Since December 2019, the coronavirus disease 2019 (COVID-19) pandemic caused by severe acute respiratory syndrome coronavirus 2 (SARS-CoV-2) has spread throughout the world and caused damage to the global economy and individual health. SARS-CoV-2 is an airborne virus (Jiang et al., 2020) and humans are primarily infected by oral and nasal inhalation. Following SARS-CoV-2 infection, typical clinical symptoms include headache, cough, fever, sore throat, fatigue, myalgia and dyspnea, which may result in death (Jiang et al., 2020; Singhal, 2020). Compared with severe acute respiratory syndrome coronavirus (SARS-CoV) and Middle East respiratory syndrome coronavirus (MERS-CoV), SARS-CoV-2 is highly infectious, and people are generally susceptible to infection by this virus. Although mortality associated with COVID-19 is low, this virus still poses a significant threat to human health (Reynolds et al., 2021). As of July 2021, it has infected over 195 million people and caused over 4.2 million deaths worldwide (Zhou et al., 2020). Therefore, there is an urgent need to understand the molecular mechanisms underlying SARS-CoV-2 pathogenesis, immune evasion, and disease progression.

SARS-CoV-2 is a beta coronavirus that belongs to the Coronaviridae family (Wu et al., 2020). It is a spherical, encapsulated, positive-sense single-stranded RNA virus. The total length of the genome is approximately 30 kb and the open reading frame (ORF) is predicted to contain 11 genes, encoding approximately 20 functional proteins (Wu et al., 2020). The genomic sequence of SARS-CoV-2 exhibits the typical structural characteristics of coronaviruses, including a 5′ untranslated region, 5′ replicase polysaccharide protein gene (ORF1/ab), spike glycoprotein gene (S), envelope glycoprotein gene (E), membrane glycoprotein gene (M), nucleocapsid protein gene (N), and 3′ untranslated region (Zhou et al., 2020; Thye et al., 2021). Among the different mutants of SARS-CoV-2 and even among different coronaviruses, the SARS-CoV-2 nonstructural protein 13 (CoV-2 Nsp13) and SARS-CoV-2 nucleocapsid protein (CoV-2 N) sequences are highly conserved (Supplementary Figure 1). Elucidating the structure and function of CoV-2 Nsp13 and CoV-2 N will be useful for the development of anti-SARS-CoV-2 drugs (Gurung, 2020; Gussow et al., 2020; Mirza and Froeyen, 2020).

CoV-2 Nsp13 is predicted to contain 596 amino acids (located in the ORF1ab polyprotein from amino acids 5,325 to 5,925; Gurung, 2020). Similar to SARS-CoV and MERS-CoV Nsp13, CoV-2 Nsp13 has a triangular, pyramid shape that consists of five domains. The structure of the pyramid-shaped CoV-2 Nsp13 consists of two RecA-like helicase subdomains (1A and 2A), which form the triangular base, an N-terminal zinc-binding domain, a helical “stalk” domain, and a beta-barrel 1B domain (Mirza and Froeyen, 2020; Newman et al., 2021). The same structural characteristics were previously reported for the SARS-CoV and MERS-CoV Nsp13 proteins (Mirza and Froeyen, 2020; Newman et al., 2021). CoV-2 Nsp13 is important for viral replication as a helicase that unwinds duplex RNA and a 5′-triphosphatase that is likely involved in the 5′-capping of viral mRNA (Min et al., 2021). Studies have found that CoV-2 Nsp13 strongly inhibits type I interferon signaling (Lei et al., 2020; Xia et al., 2020), highlighting the versatile activities of CoV-2 Nsp13 during viral infection. Another study reported that SARS-CoV-2 blocks immune activation during infection, suggesting that CoV-2 Nsp13 plays a role in blocking IFN and NF-κB activation and acts as an immune regulator (Vazquez et al., 2021). Studies have indicated that CoV-2 Nsp13 exhibits 5′-3′ unwinding activity on double-stranded DNA (dsDNA) and double-stranded RNA (Mickolajczyk et al., 2021).

CoV-2 N consists of 413 amino acid residues and is the only protein that binds to genomic RNA in the nucleocapsid (Kang et al., 2020). It is involved in viral replication and cell signaling pathway regulation (Carlson et al., 2020). CoV-2 N is one of the most conserved proteins in coronaviruses (Supplementary Figure 1; McBride et al., 2014). CoV-2 N of various coronaviruses exhibits high immunogenicity, which can induce the body to produce a robust immune response (Aboagye et al., 2018; Smits et al., 2021). CoV-2 N has been repeatedly proposed as a vaccine candidate, suggesting that it has the potential to induce an immune response capable of preventing infection from various strains of human coronaviruses (Yang et al., 2009; Shi et al., 2015). To develop drug targets for COVID-19, Gussow et al. used a novel approach combining advanced machine learning methods and traditional genome comparison technology to screen four potential key regions in coronavirus strains that result in high mortality rates and identify potential genomic determinants, three of which are located in CoV-2 N. Thus, CoV-2 N may be a key target in combating the COVID-19 pandemic (Gussow et al., 2020). Therefore, it is particularly important to study the biochemical function of CoV-2 N, which will provide insights on future vaccine research.

Initially, the N protein was known as an RNA-binding protein critical for viral genome packaging. Owing to its distinct RNA-binding activity, it was recently reported to bind to host mRNAs and further interfere with the normal functioning of the host (Nabeel-Shah et al., 2022). Because this protein can also bind to DNA nonspecifically (Tang et al., 2005; Zhao et al., 2021), it may also perform specific functions by binding to DNA in cells. Interestingly, the N protein of human immunodeficiency virus, also a positive-sense single-stranded RNA virus, performs cellular functions by binding to DNA (Gien et al., 2022). However, little is known about the dynamics of the interaction between the N protein and DNA.

The ORF of SARS-CoV-2 is predicted to encode approximately 20 functional proteins. Among these, only CoV-2 Nsp13 has been reported to exhibit helicase activity. Surprisingly, we found that the SARS-CoV-2 N protein (a structural protein) can open double-stranded nucleic acids. However, the two proteins differ to a considerable extent in sequence and structure, which suggests that their mechanisms of opening nucleic acid substrates are different (Supplementary Figure 2). This biochemical characteristic of CoV-2 N is similar to that of single-stranded binding proteins, which exist widely. In prokaryotes, the representative protein is *Escherichia coli* single-stranded DNA (ssDNA)-binding protein (SSB), whereas in eukaryotes, the representative protein is replication protein A (RPA), both of which exhibit dsDNA unwinding activity (Wang et al., 2004; Safa et al., 2016). Of these, RPA, which is an essential factor in DNA metabolism, can unwind dsDNA directly or initially combine with dsDNA and recruit other proteins to unwind dsDNA (Fan et al., 2009).

To gain insights on the various functions of CoV-2 N, we expressed and purified full-length CoV-2 N protein and compared the dsDNA unwinding activities of CoV-2 N and CoV-2 Nsp13. We demonstrated that recombinant CoV-2 N exhibits efficient unwinding activity with several DNA substrates involved in DNA replication, repair, and recombination. Although CoV-2 N can unwind dsDNA, it does not possess the characteristics of a typical helicase. Interestingly, CoV-2 N strongly promotes ssDNA annealing at low concentrations, whereas it exhibits unwinding activity at high concentrations.

## Materials and Methods

### Reagents and Buffers

All chemicals were of reagent grade. Buffers were prepared using high-quality deionized water from a Milli-Q ultrapure water purification system (Millipore, Burlington, MA, United States) with a resistivity greater than 18.2 MΩ·cm and further filtered through a 2-μm filter before use. All chemicals were purchased from Sigma (St. Louis, MO, United States) unless otherwise indicated. All solutions were filtered and extensively degassed immediately before use.

### Preparation of DNA

The DNA substrates used in unwinding and annealing experiments as well as the primers were purchased from Shanghai Sangon Biological Engineering Technology & Services Co., Ltd. (Shanghai, China). Various substrate structures were designed such as DNA with different 5′ and 3′ overhang lengths and various nucleotide length internal bubbles. The 3′ or 5′ end of one of the ssDNAs was fluorescence (FAM) labeled so that single- and double-stranded changes could be observed at a wavelength of 560 nM. Details of the structures and sequences of unlabeled or FAM-labeled DNA substrates are shown in Table 1. Duplex substrates were annealed by incubating at 95°C for 5 min, followed by cooling to 25°C for approximately 7 h [annealing buffer: 25 mM Tris–HCl (pH 7.5) and 50 mM NaCl]. The various duplex substrates were stored at −20°C.

**Table 1:** Sequences and structure information of the unwinding and annealing substrates.

Name	Structure	Sequence(F,Fluorescein)	Comment
5′-OhS22D21	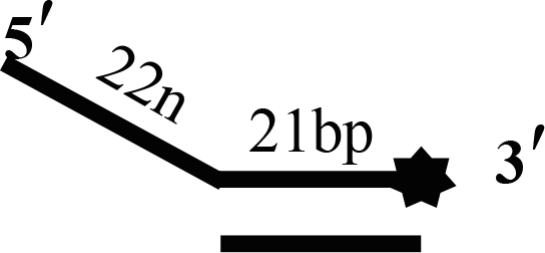	5′-CTGTAGGAATGTGAAATAAAAACGATGTTTTATTTACATTGTA-3′-F 3′-GCTACAAAATAAATGTAACAT-5′	5′-22 nt-Overhanged-21 bp
S43	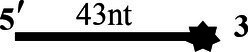	5′-CTGTAGGAATGTGAAATAAAAACGATGTTTTATTTACATTGTA-3′-F	43 nt ssDNA
S21	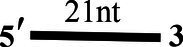	3′-GCTACAAAATAAATGTAACAT-5′	21 nt ssDNA
3′-OhS22D21	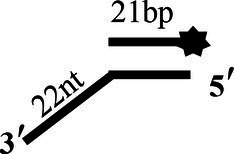	3′-CTGTAGGAATGTGAAATAAAAACGATGTTTTATTTACATTGTA-5′ 5′-GCTACAAAATAAATGTAACAT-3′-F	3′-22 nt-Overhanged-21 bp
S43-a	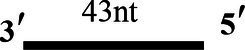	3′-CTGTAGGAATGTGAAATAAAAACGATGTTTTATTTACATTGTA-5′	43 nt ssDNA
S21-a	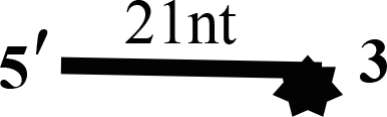	5′-GCTACAAAATAAATGTAACAT-3′-F	21 nt ssDNA
DS32	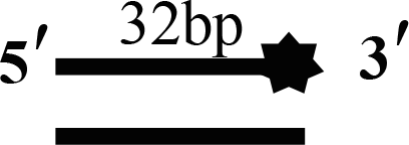	5′-TATCGAAGAATGTTATGTCATTCCGGCAGATG-3′-F 3′-ATAGCTTCTTACAATACAGTAAGGCCGTCTAC-5′	32 bp dsDNA
5′-OhS4D20	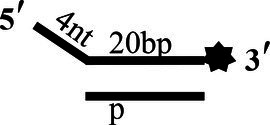	5′-AATGTTATGTCATTCCGGCAGATG-3′-F 3′-AATACAGTAAGGCCGTCTAC-5′	5′-4 nt-Overhanged-20 bp
5′-OhS12D20	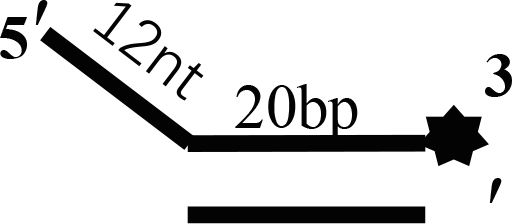	5′-TATCGAAGAATGTTATGTCATTCCGGCAGATG-3′-F 3′-AATACAGTAAGGCCGTCTAC-5′	5′-12 nt-Overhanged-20 bp
5′-OhS14D20	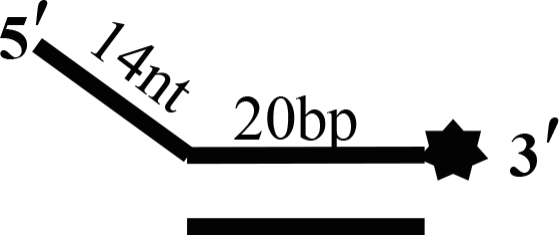	5′-CCTATCGAAGAATGTTATGTCATTCCGGCAGATG-3′-F 3′-AATACAGTAAGGCCGTCTAC-5′	5′-14 nt-Overhanged-20 bp
5′-OhS15D20	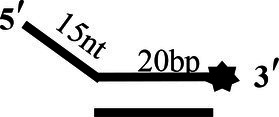	5′-TCCTATCGAAGAATGTTATGTCATTCCGGCAGATG-3′-F 3′-AATACAGTAAGGCCGTCTAC-5′	5′-15 nt-Overhanged-20 bp
5′-OhS16D20	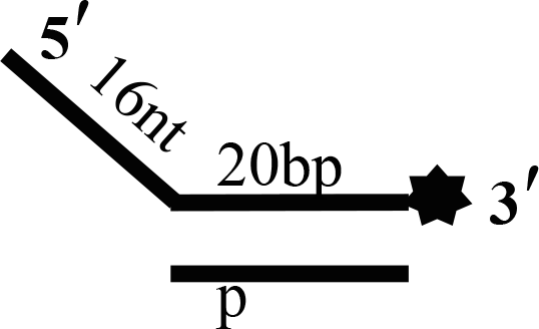	5′-ATCCTATCGAAGAATGTTATGTCATTCCGGCAGATG-3′-F 3′-AATACAGTAAGGCCGTCTAC-5′	5′-16 nt-Overhanged-20 bp
BS4	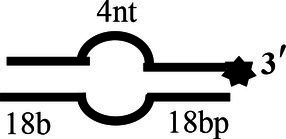	5′-CCATGCAGCTGTCAGTCCATTGTCATGCTAGGCCTACTGC-3′-F 3′-GGTACGTCGACAGTCAGGATTGAGTACGATCCGGATGACG-5′	Bubble-4 nt
BS12	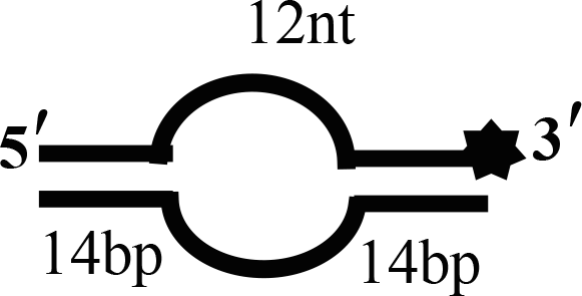	5′-CCATGCAGCTGTCAGTCCATTGTCATGCTAGGCCTACTGC-3′-F 3′-GGTACGTCGACAGTGTCCATTGTCATCGATCCGGATGACG-5′	Bubble-12 nt
BS14	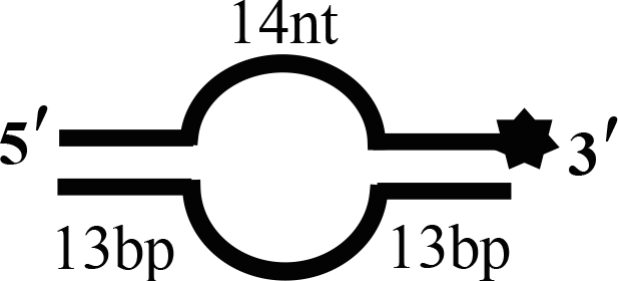	5′-CCATGCAGCTGTCAGTCCATTGTCATGCTAGGCCTACTGC-3′-F 3′-GGTACGTCGACAGAGTCCATTGTCATGGATCCGGATGACG-5′	Bubble-14 nt
BS15	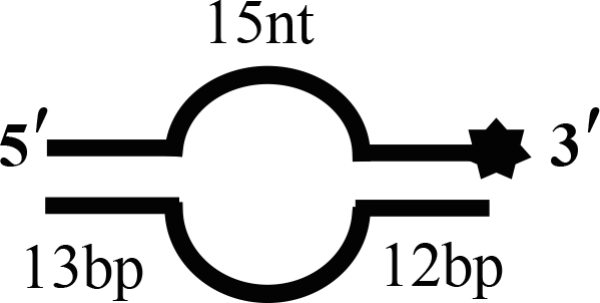	5’-CCATGCAGCTGTCAGTCCATTGTCATGCTAGGCCTACTGC-3’-F 3’-GGTACGTCGACAGAGTCCATTGTCATGCATCCGGATGACG-5’	Bubble-15 nt
BS16	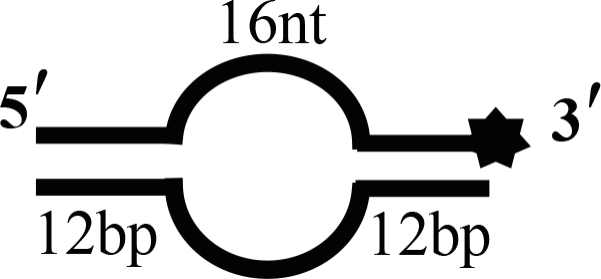	5’-CCATGCAGCTGTCAGTCCATTGTCATGCTAGGCCTACTGC-3’-F 3’-GGTACGTCGACACAGTCCATTGTCATGC ATCCGGATGACG-5’	Bubble-16 nt

### Recombinant Plasmids

Plasmid pET-28a-2019-nCoV-N was obtained from Guangdong Laboratory Animal Monitoring and contained the CoV-2 N coding sequence (CoV-2 N GenBank accession: NC_045512.2), which has a 6xHis-tag cloned downstream of the AUG promoter. pSmart-I-CoV-2 Nsp13 contained the CoV-2 Nsp13 coding sequence (CoV-2 Nsp13 GenBank accession: OM019196.1) with a 6-his tag and an in-frame N-terminal SUMO fusion tag cloned downstream of the AUG promoter in the pSmart-I expression vector (Jing et al., 2016). The latter is a SUMO protease-cleavage site between the SUMO tag and the expressed protein. The pSmart-I-CoV-2 Nsp13 was synthesized by General Biosystems Co., Ltd. (Anhui, China).

### Protein Expression and Purification

The pET28a-CoV-2 N and pSmart-I-CoV-2 Nsp13 vectors were transformed into *E. coli* 2,566 for protein expression. The cells were grown in LB medium and the proteins were expressed overnight at 18°C after induction with 0.3 mM and 0.6 mM isopropyl β-d-1-thiogalactopyranoside (IPTG), respectively. All media contained 50 μg/ml kanamycin. The supernatant containing CoV-2 N was precipitated with 3.5 M ammonium sulfate for 3 h. After removing the supernatant, the precipitate was redissolved in a buffer containing 20 mM Tris–HCl (pH 7.5) and 300 mM NaCl. The supernatant was loaded onto Ni-NTA Sepharose beads (GE Healthcare, Boston, United States). The protein was eluted with 200 mM imidazole, followed by reducing the concentration of NaCl to 50 mM by dialysis. The protein was further purified on the SP Sepharose 6 Fast Flow column (GE Healthcare, Boston, United States). The eluted fractions were collected and concentrated, following which 5% glycerol (v/v) was added to them. The preparation was subsequently stored at −80°C.

The cleared lysate containing CoV-2 Nsp13 with a SUMO tag was loaded onto Ni-NTA Sepharose beads (GE Healthcare, Boston, United States). The protein was eluted with 200 mM imidazole. Fractions containing the proteins of interest were pooled and dialyzed overnight at 4°C against a buffer containing 25 mM Tris–HCl (pH 7.5) and 500 mM NaCl. The constructs were cleaved with SUMO protease [1:100 SUMO protease: protein (molar ratio)] to remove the SUMO tag. After dialysis, the samples were reapplied to Ni-NTA Sepharose beads using the same purification buffers. The solution containing CoV-2 Nsp13 was precipitated using 3.5 M ammonium sulfate for 3 h. After removing the supernatant, the precipitate was redissolved in a buffer containing 20 mM Tris–HCl (pH 7.5) and 300 mM NaCl. After redissolving, the supernatant was further purified by gel filtration chromatography using a SuperDeX 200 10/300 GL column (GE Healthcare, Boston, United States) and a buffer containing 20 mM Tris–HCl (pH 7.5), 300 mM NaCl, 10% glycerol (v/v), and 2 mM dithiothreitol (DTT). The eluted fractions were collected, concentrated, and stored at −80°C.

### Unwinding and Annealing Assays

CoV-2 N was incubated with dsDNA in unwinding buffer A [25 mM Tris–HCl (pH 7.5) and 300 mM NaCl] at 30°C for 10 min. CoV-2 Nsp13 was incubated with dsDNA in unwinding buffer B [25 mM Tris–HCl (pH 7.5), 50 mM NaCl, 1.5 mM MgCl_2_, and 1 mM DTT], and 10 mM adenosine triphosphate (ATP) was added to initiate the reaction at 30°C for 10 min. The reactions were quenched by the addition of 5× stop loading buffer (150 mM EDTA, 2% SDS, 30% glycerol, and 0.1% bromophenol blue). The products of the DNA unwinding reactions were resolved on native 12% PAGE gels (Acr:Bis = 39:1) at 100 V for 80 min. The DNA in the polyacrylamide gels was visualized using ChemiDoc MP and quantitated using Image Lab software (Bio-Rad, California, United States).

Two DNA substrates were used for the DNA annealing assays. Substrate S1 was 43 nt (nucleotide) and substrate S2 was 21 nt (nucleotide). CoV-2 N was incubated with the two single DNAs in buffer A (the same as unwinding buffer A) at 30°C for 10 min. CoV-2 Nsp13 was incubated with two the single DNAs in buffer B (the same as unwinding buffer B) at 30°C for 10 min. The reactions were quenched by the addition of 5 × stop loading buffer. The products of the DNA annealing reactions were then resolved on native 12% PAGE gels (Acr:Bis = 39:1) at 100 V for 80 min. The DNA in the polyacrylamide gels was visualized using ChemiDoc MP (Bio-Rad).

### Binding Assay

The fluorescently labeled DNA substrate can rotate freely when it is not bound to the enzyme molecule (i.e., showing low fluorescence anisotropy). When the substrate binds to the enzyme molecule to form a complex, its free rotation is significantly reduced, resulting in a significant increase in fluorescence anisotropy. Therefore, changes in anisotropy indirectly reflect the combined state of the DNA substrate and protein. The fluorescently labeled DNA substrate was complexed with varying amounts of protein in binding buffer [CoV-2 N: 50 mM Tris–HCl and 300 mM NaCl (pH 7.0); Nsp13: 50 mM Tris–HCl and 20 mM NaCl (pH 7.0)]. Each sample was allowed to equilibrate in solution for 5 min, following which steady-state fluorescence anisotropy was measured. A second reading was taken after 10 min to ensure that the mixture was well equilibrated and stable. As detected using the SpectraMax iD3 microplate reader (Molecular Devices, LLC, United States), the rotation decreases as its anisotropy increases. The equilibrium dissociation constant was determined by fitting the binding curves using Equation 1:


(1) $$\Delta r=\Delta r_{\max}\times P÷\left(k_{d}+P\right)$$

where Δr_max_ is the maximal amplitude of anisotropy (=r_max_, RNA/DNA–protein complex − r_free_ RNA/DNA), *P* is protein concentration, and K_d_ represents the equilibrium dissociation constant, which is the corresponding protein concentration when it reaches half of the maximum anisotropy in the fitted curve.

## Results

### Expression and Purification of CoV-2 N and CoV-2 Nsp13

**Figure 1:**
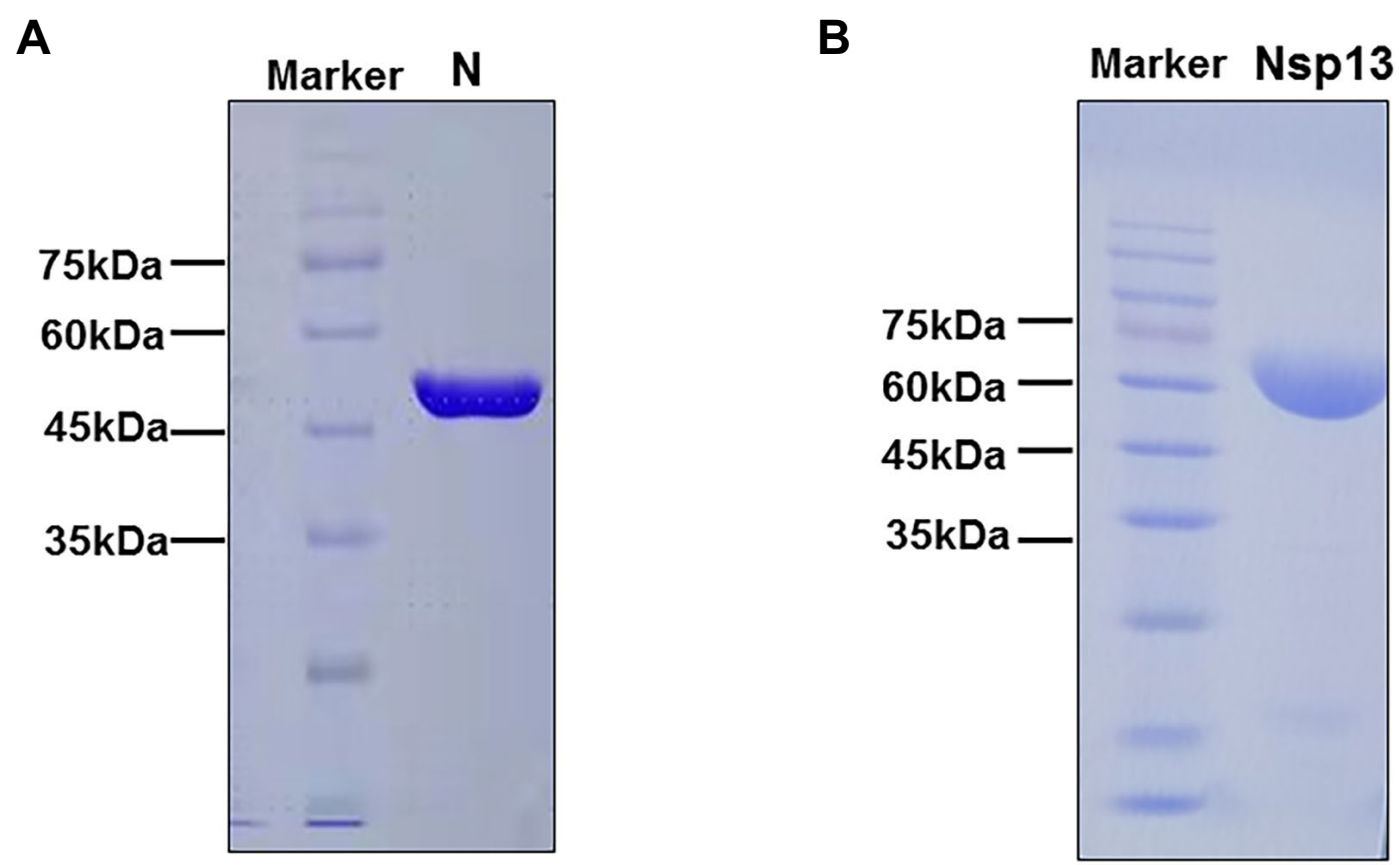
SDS-PAGE analysis of purified CoV-2 N and CoV-2 Nsp13 preparations. **(A,B)** Approximately 25 μg of purified CoV-2 N and CoV-2 Nsp13 were electrophoresed on 10% polyacrylamide gels and stained with Coomassie Brilliant Blue. The positions of the molecular weight (MW) markers are shown on the left of each image. The molecular weights of CoV-2 N and CoV-2 Nsp13 were approximately 47 kDa and 68 kDa, respectively. Lane N: CoV-2 N; Lane Nsp13: CoV-2 Nsp13.

To analyze the biochemical characteristics of recombinant CoV-2 N and CoV-2 Nsp13, we first constructed and purified them. SDS-PAGE revealed that the molecular weights of CoV-2 N and CoV-2 Nsp13 were 47 kDa and 68 kDa, respectively, which are consistent with the theoretical molecular weights (Figures 1A,B).

### CoV-2 N Unwinding Has a Polarity of 5′–3′

**Figure 2:**
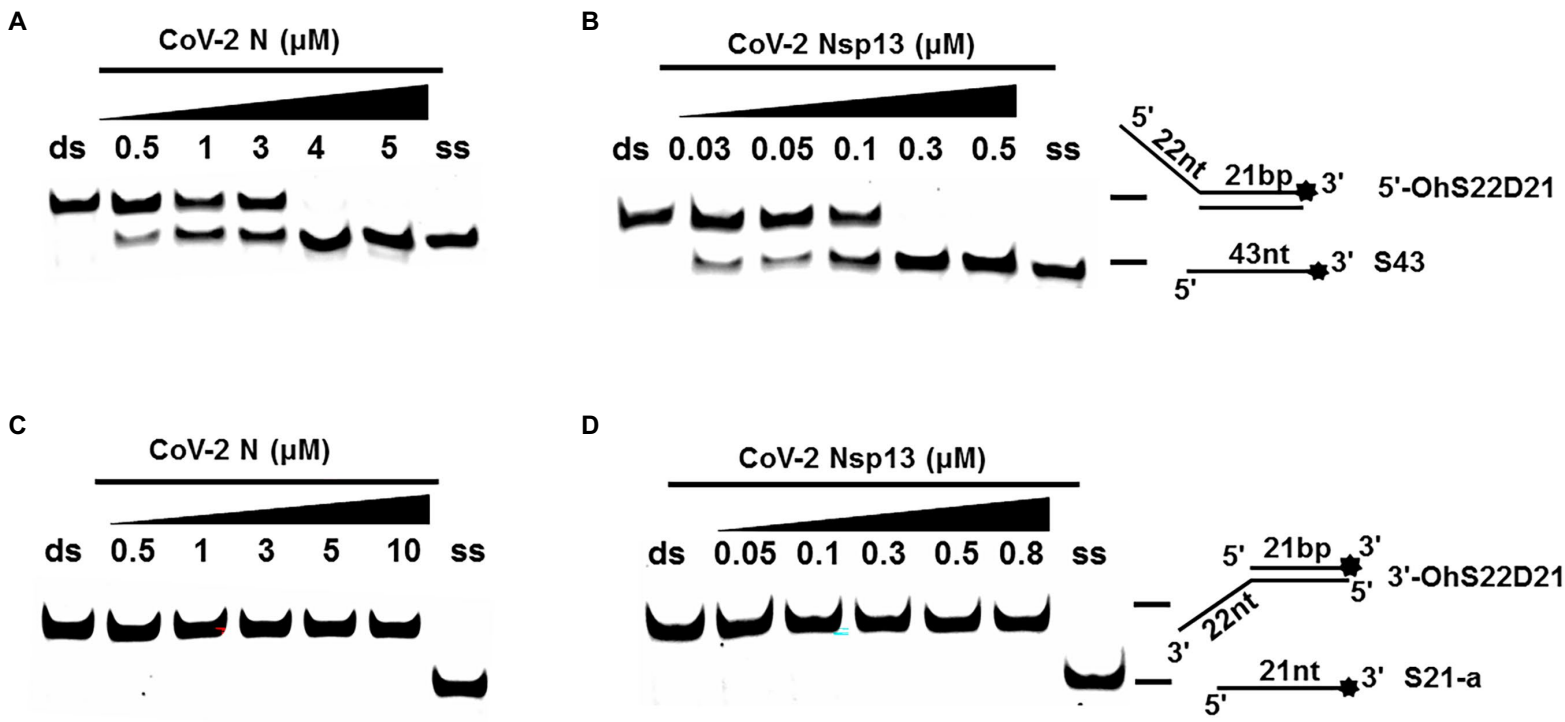
Unwinding polarity of CoV-2 N and CoV-2 Nsp13. **(A,B)** CoV-2 N and CoV-2 Nsp13 unwind dsDNA containing a 5′-overhang (5′-OhS22D21). **(C,D)** CoV-2 N and CoV-2 Nsp13 cannot unwind dsDNA with a 3′-overhang (3’-OhS22D21). All experiments were performed under standard experimental conditions. dsDNA (5′-OhS22D21 or 3’-OhS22D21) was assayed at 10 nM and contained a fluorescent label on the 3′ end, and protein concentration gradually increased. Lane ds: duplex -DNA; Lane ss: single DNA with 3′-fluorescein (3’-FAM; shown in Table 1). Top of the arrow: duplex DNA is not unwound; Bottom of the arrow: unwound single-stranded DNA. Native PAGE (Acr:Bis = 39:1; 12%), 100 V, 80 min. The fluorescence signals were visualized using a ChemiDoc MP Imaging System (Bio-Rad, California, United States). The detailed experimental conditions for the unwinding assay are described in the Materials and Methods section.

One of the important characteristics of dsDNA unwinding is the unwinding polarity, which is defined by the backbone polarity of the flanking ssDNA that promotes the initiation of unwinding and defines, in turn, the direction of helicase movement along the strand. To determine and compare the polarity of CoV-2 N and CoV-2 Nsp13, partial dsDNA substrates with either a 5′or 3′ single-stranded overhang were used (5’-Oh S22D21 and 3’-OhS22D21, respectively, Table 1). The results clearly showed that CoV-2 N and CoV-2 Nsp13 had similar unwinding polarities. They unwound the 5′ single-stranded overhang–containing substrate only (Figures 2A,B) and not the 3′ single-stranded overhang–containing substrate (Figures 2C,D).

Complete unwinding of the dsDNA required 5 μM CoV-2 N but only 0.3 μM CoV-2 Nsp13 (Figures 2A,B). Thus, the unwinding activity of CoV-2 N was more than 22 times weaker than that of CoV-2 Nsp13 (Supplementary Figures 3A–C). We also compared the Km value of the unwinding reaction, which is one of the most important parameters for evaluating the activity of helicases (Supplementary Figure 3D).

### CoV-2 N Is Not a Helicase

**Figure 3:**
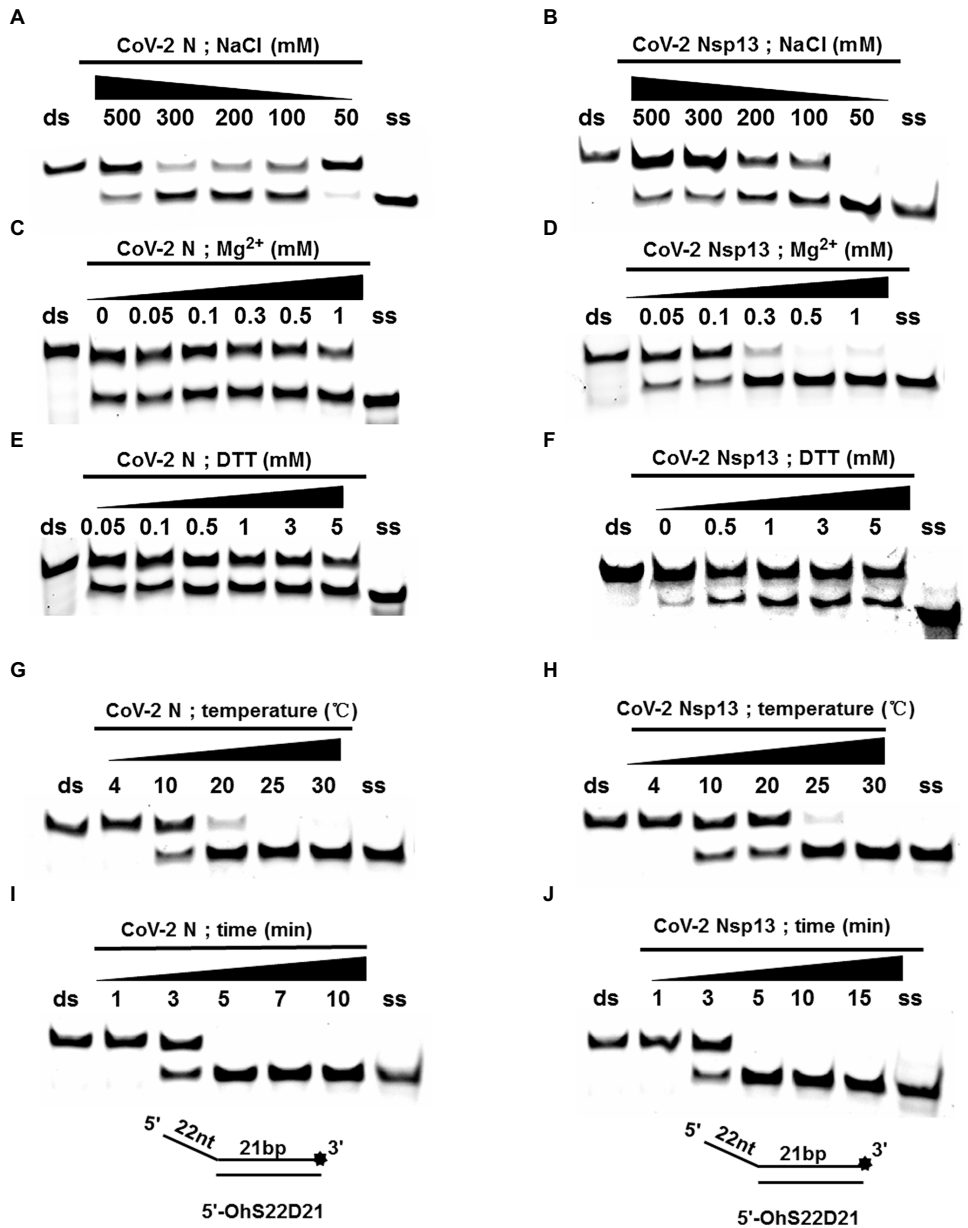
Determination and comparison of optimal buffer compositions for CoV-2 N and CoV-2 Nsp13 unwinding. **(A–F)** Protein concentrations of CoV-2 N and CoV-2 Nsp13 were 3 μM (note: the protein concentration is 5 μM in **A**) and 0.3 μM (note: the protein concentration is 0.1 μM in **F**), respectively, and the reactions were performed in buffer A and buffer B (removing the single-factor NaCl, Mg^2+^, DTT shown in **A–F**). **(G–J)** The protein concentrations of CoV-2 N and CoV-2 Nsp13 were saturating concentrations of 5 μM and 0.3 μM, respectively. All solutions used were kept on ice for precooling. Samples were placed on ice before reactions were initiated. A water bath of the corresponding temperature was used immediately after reactions were started, and stop buffer was added at appropriate times to terminate reactions. All used substrates were 5′-overhang DNA (5’-OhS22D21) at 10 nM.

Using an *in vitro* gel assay system, the unwinding activity of recombinant CoV-2 N and CoV-2 Nsp13 was examined. To determine and compare the conditions for optimal nucleotide unwinding activity, we evaluated different concentrations of protein, NaCl, DTT, and divalent cations (Mg^2+^), and compared the unwinding temperature and time using a substrate containing a 5′ single-stranded overhang (5′-OhS22D21, Table 1). A typical helicase usually binds more efficiently to nucleic acid substrates at low salt concentrations, which also promote the activity of the helicase. CoV-2 N showed a better ratio of unwinding at 100–300 mM NaCl, whereas CoV-2 Nsp13 exhibited a stronger unwinding ability as NaCl concentration decreased, causing complete unwinding at 50 mM NaCl (Figures 3A,B). Helicases are motor proteins that hydrolyze ATP for energy in a process that requires divalent metal ions, such as Mg^2+^. We adjusted the protein concentration to 3 μM, and the ratio of unwinding at this concentration in the reaction without Mg^2+^ was approximately 50% (Figure 3C). The addition of Mg^2+^ did not considerably inhibit or promote the unwinding activity of CoV-2 N, and the unwinding ratio was similar to that in the reaction without Mg^2+^, indicating that CoV-2 N does not require Mg^2+^ to unwind dsDNA (Figure 3C). However, CoV-2 Nsp13 showed a strong dependence on Mg^2+^ for dsDNA unwinding (Figure 3D). Notably, the unwinding activity of CoV-2 N was independent of DTT as the addition of DTT to the reaction mixture had no obvious effects on unwinding (Figure 3E). However, CoV-2 Nsp13 showed dependence on DTT, exhibiting an optimum response at 1–3 mM DTT (Figure 3F). Temperature is also a crucial factor that affects unwinding activity. Assays for *in vitro* helicase activity are usually performed at 4–30°C. The dsDNA was completely unwound by CoV-2 N when the reaction was performed at 25°C–30°C (Figure 3G), whereas it was unwound by CoV-2 Nsp13 only at 30°C (Figure 3H). Finally, we analyzed the time required for CoV-2 N and CoV-2 Nsp13 to completely unwind the dsDNA (5′-OhS22D21). Similar to the temperature experiment, the whole reaction process was performed at low temperature. The samples were removed at the corresponding reaction times (Figures 3I,J), and stop buffer was added immediately. The results indicated that the unwinding rate of the two was similar. After 5 min of initiating the reaction, CoV-2 N and CoV-2 Nsp13 had almost completely unwound the dsDNA (Figures 3I,J).

### CoV-2 N Unwinding Activity Does Not Depend on Nucleoside Triphosphates

A typical helicase usually not only relies on ATP but also utilizes other nucleoside triphosphates (NTPs), such as cytidine triphosphate (CTP), guanine triphosphate (GTP), and uridine triphosphate (UTP). To determine the effect of NTPs on the unwinding activity of CoV-2 N, we set the final concentration of the reaction of CoV-2 N to 3 μM in the reaction. At this concentration, dsDNA was unwound to approximately 50% in the absence of NTPs (Figure 2A).

**Figure 4:**
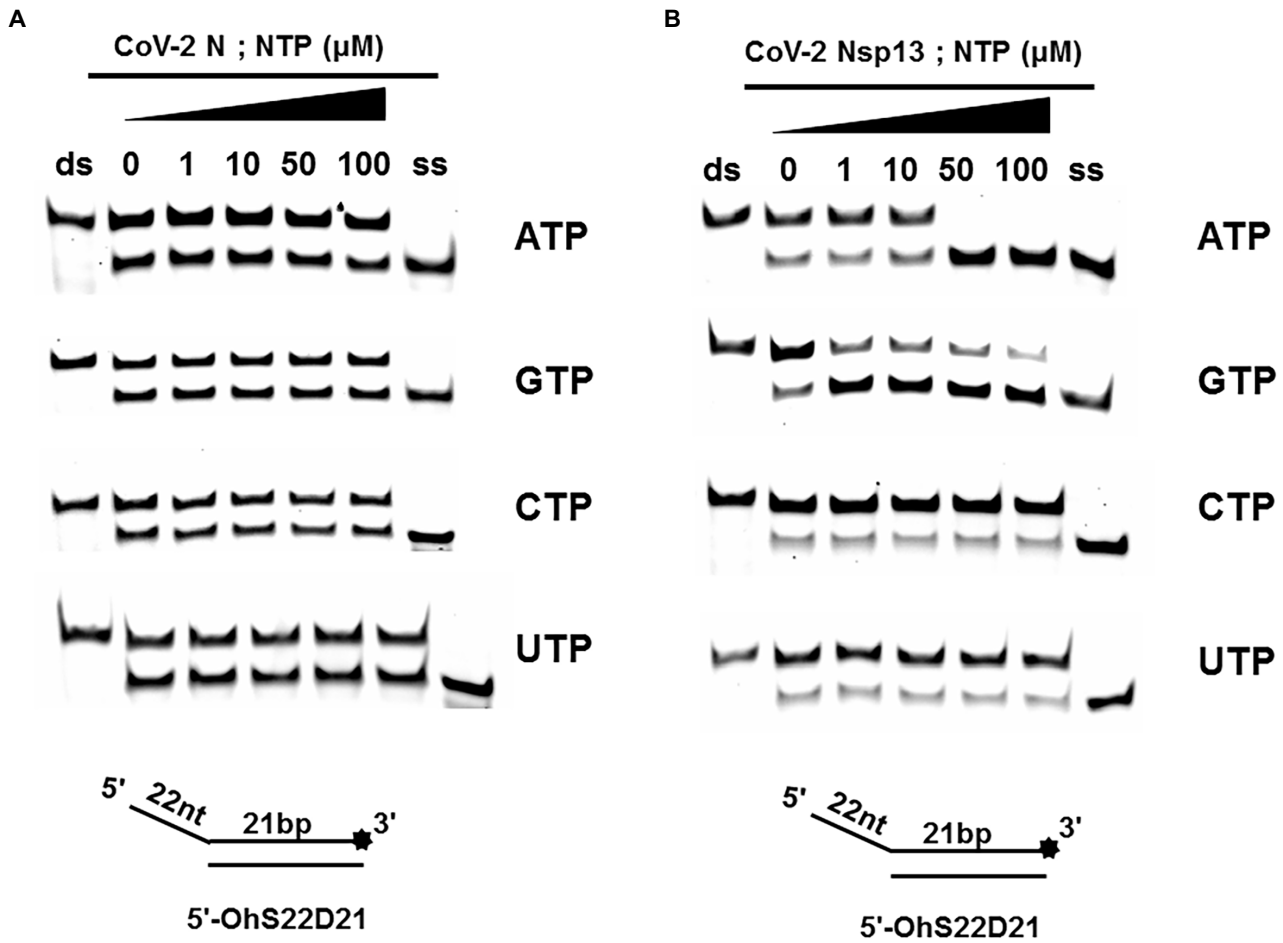
Unwinding activities of CoV-2 N and CoV-2 Nsp13 in the presence of different nucleoside triphosphates at different concentrations. **(A,B)** The 5′-overhang DNA substrate at 10 nM (5’-OhS22D21). CoV-2 N concentration was 3 μM in buffer A and CoV-2 Nsp13 was 0.3 μM in buffer B, respectively. For a clearer analysis of the propensity of CoV-2 Nsp13 to NTP species, we performed comparisons by scanning gray values using Image Lab software (Supplementary Figure 4). All experiments were performed under experimental conditions described in “Materials and Methods.”

The results showed that CoV-2 N could still unwind the dsDNA (5′-Oh S22D21) in reaction buffer A, which did not contain NTP, and a gradual increase in NTP concentration had no obvious effect on the promotion or inhibition of unwinding (Figure 4A). The opening of double-stranded nucleic acids by CoV-2 N does not appear to be dependent on the energy provided by ATP hydrolysis. It is more likely that CoV-2 N binds to the transiently opened single-stranded nucleic acid (similar to SSB and RPA) to prevent complementary base repairing.

Unlike CoV-2 N, CoV-2 Nsp13 is a typical helicase, and ATP usually participates in the unwinding process. Therefore, we set the final concentration of CoV-2 Nsp13 to 0.3 μM, which was sufficient to fully unwind the double-strand nucleic acid present in the reaction mixture at saturation concentration. The results indicated that although CoV-2 Nsp13 could still partially unwind dsDNA in reaction buffer B, we observed that in samples containing ATP and GTP, the unwinding ratio increased with increasing concentrations of ATP and GTP (Figure 4B). We compared the unwinding ratios of NTPs at 10 μM and 50 μM, respectively, to demonstrate the ability of CoV-2 Nsp13 (0.3 μM) to utilize the four NTPs (Supplementary Figure 4).

### Unwinding Activity of CoV-2 N and CoV-2 Nsp13

**Figure 5:**
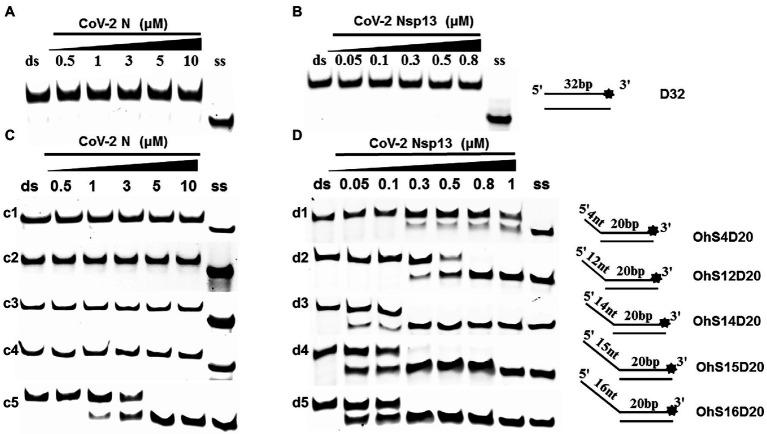
Comparison of the effect of single-strand overhang length on the unwinding ability of CoV-2 N and CoV-2 Nsp13. **(A,B)** Blunt-end dsDNA substrate (DS32). **(C,D)** Comparison of the effect of single-strand overhang length (4 nt–16 nt) on the unwinding activities of CoV-2 N and CoV-2 Nsp13. (c1, d1) 5’-OhS4D20; (c2, d2) 5’-OhS12D20; (c3, d3) 5’-OhS14D20; (c4, d4) 5’-OhS15D20; (c5, d5) 5’-OhS16D20. The unwinding activities of CoV-2 N and CoV-2 Nsp13 were assayed in the presence of increasing protein concentrations in buffer A and buffer B, respectively. All 5′-overhang dsDNA substrates used at 10 nM.

To determine the physiological functions of CoV-2 N and CoV-2 Nsp13 during viral replication, various nucleotides (D32, OhS4D20, OhS12D20, OhS14D20, OhS15D20 and OhS16D20 in Table 1) were fluorescently labeled to determine the unwinding activity of the two proteins in an *in vitro* gel assay. Neither CoV-2 N nor CoV-2 Nsp13 could unwind blunt dsDNA (Figures 5A,B). This suggests that CoV-2 N and CoV-2 Nsp13 require a 5′-ss tail to effectively unwind dsDNA.

**Figure 6:**
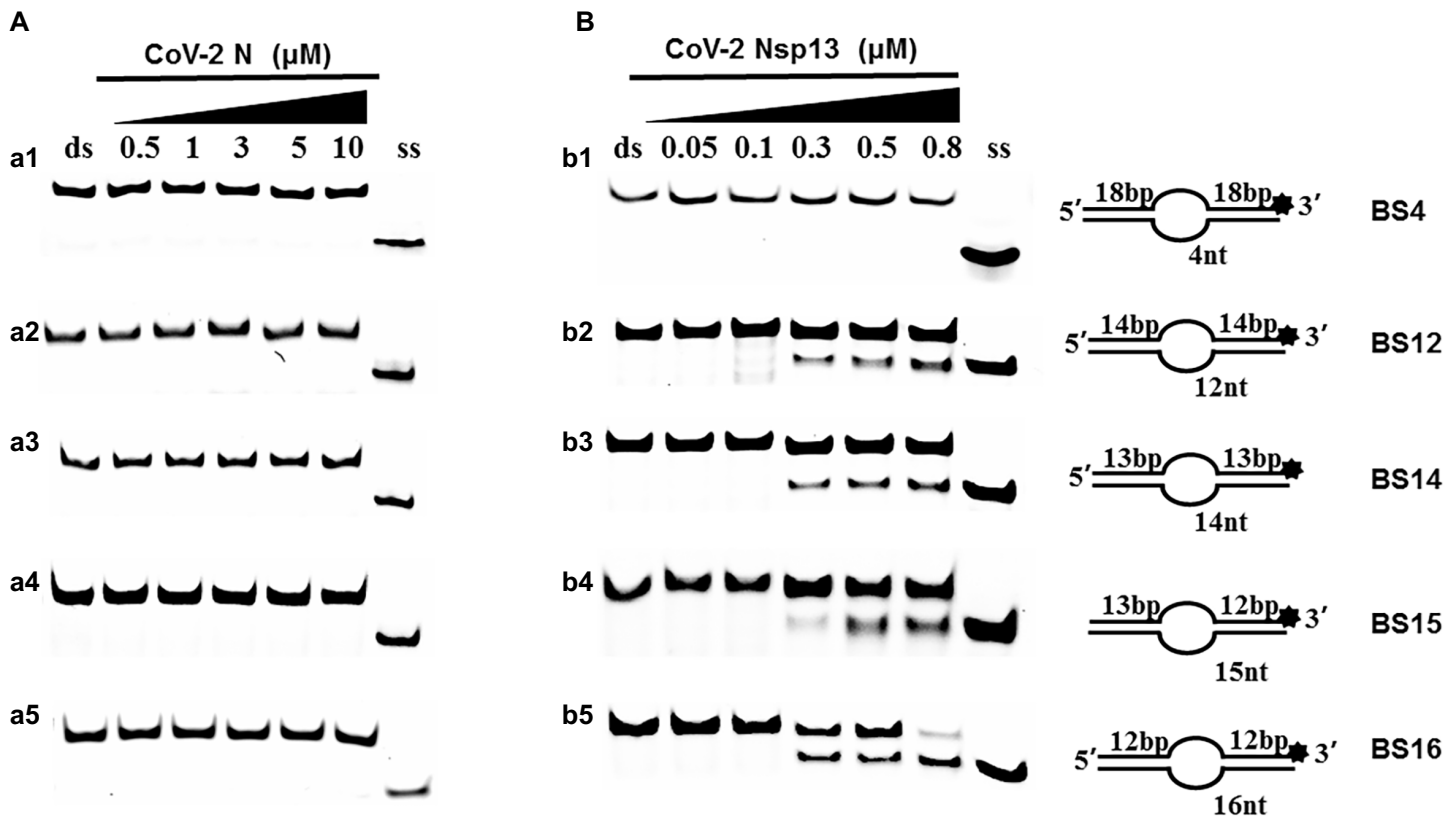
Comparison of the effect of the dsDNA bubble structure on the unwinding ability of CoV-2 N and CoV-2 Nsp13. (a1, b1) BS4; (a2, b2) BS12; (a3, b3) BS14; (a4, b4) BS15; and (a5, b5) BS16. All experiments were performed using a series of dsDNA bubble substrates (shown in Table 1), The unwinding activities of CoV-2 N and CoV-2 Nsp13 were assayed in the presence of increasing protein concentrations in buffer A and buffer B, respectively. All the bubble dsDNA substrates fixed at 10 nM. All experiments were performed under standard experimental conditions as described in “Materials and Methods.” **(A,B)** Comparison of dsDNA bubble structure (4 nt -16 nt) on the unwinding activities of CoV-2 N and CoV-2 Nsp13.

Examining the effect of single-stranded overhang length on the unwinding abilities of CoV-2 N and CoV-2 Nsp13 indicated that the overhang length of the dsDNA substrate should be at least 16 nucleotides for CoV-2 N to unwind it (Figure 5C). However, CoV-2 Nsp13 was able to unwind substrates with overhangs of ≤16 nucleotides in length, with the efficacy increasing with increasing overhang length increased; however, no obvious difference was observed when the overhang length exceeded 14 nucleotides (Figure 5D and Supplementary Figure 5).When different concentrations of CoV-2 N and CoV-2 Nsp13 were incubated with dsDNA containing various bubble structures (BS4, BS12, BS14, BS15, and BS16), CoV-2 N could not alter the structure of the dsDNA (Figure 6A); however, CoV-2 Nsp13 was able to unwind it. When the bubble spanned 12 nucleotides, the unwinding ratio improved with the length of the single-stranded (Figure 6B and Supplementary Figure 6). The dsDNA containing bubble structures is similar to a two-linked, forked substrates. Each fork structure contains a 3′ single-stranded tail. Therefore, CoV-2 Nsp13 was able to unwind both strands, which did not involve polarity.

### Annealing Activity of CoV-2 N and CoV-2 Nsp13

**Figure 7:**
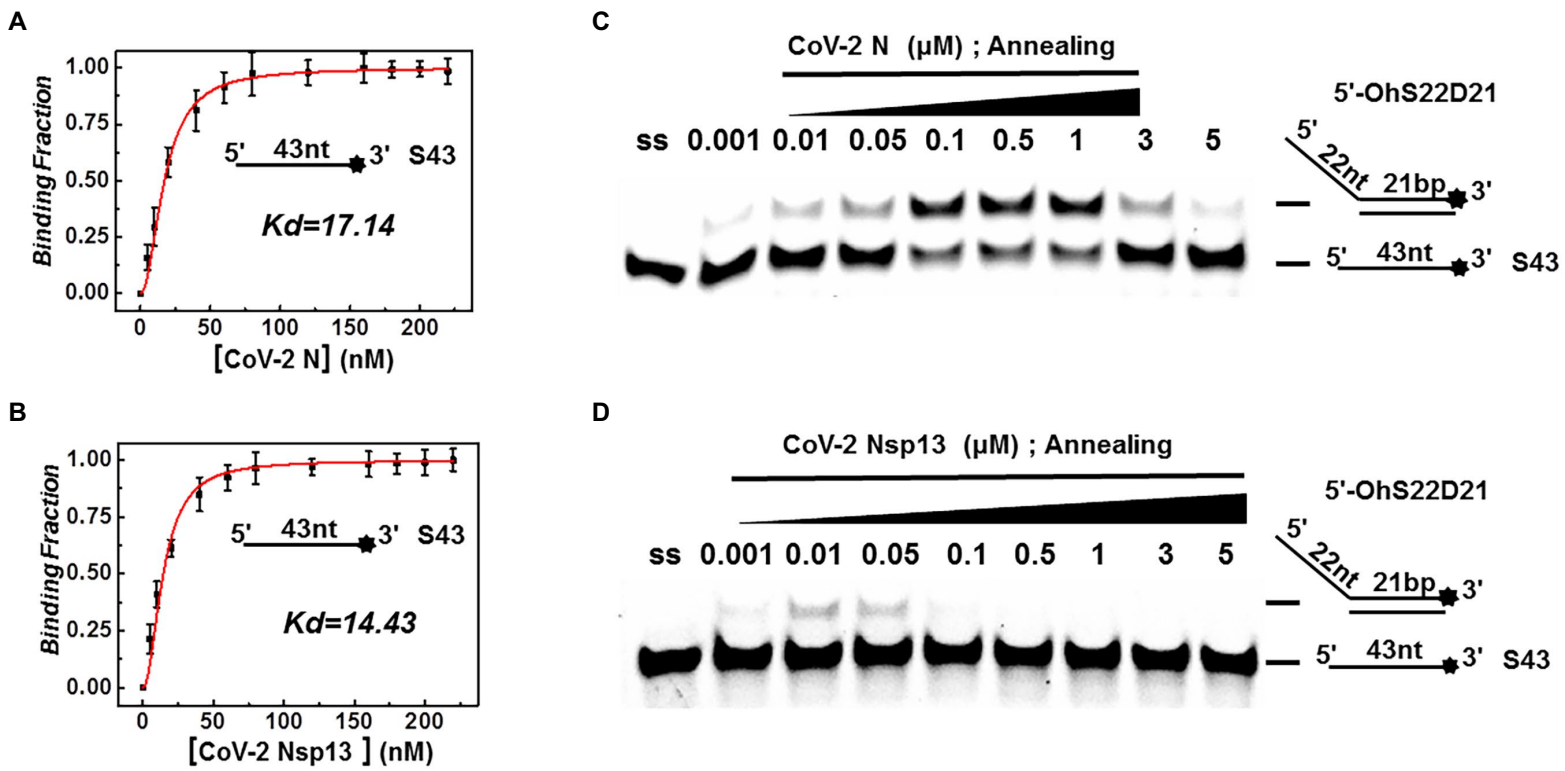
Binding and annealing activities of CoV-2 N and CoV-2 Nsp13. **(A,B)** The binding activity of single DNA FAM-labeled (S43) to CoV-2 N and CoV-2 Nsp13 was evaluated by comparing binding fractions and dissociation constants (Kd). The substrate used was ssDNA-S43 at a concentration of 5 nM, and protein concentration(s) gradually increased. All experiments were performed under standard experimental conditions as described in the Materials and Methods. **(C,D)** The annealing activities of CoV-2 N **(C)** and CoV-2 Nsp13 **(D)** were assayed in the presence of increasing protein concentrations in buffer A and buffer B at 30°C for 10 min, respectively. Native PAGE (Acr:Bis = 39:1) 12%, 100 V, 80 min. The two single-stranded DNA substrates (S43, S21) were used at 10 nM. Fluorescence signals were visualized using a ChemiDoc MP Imaging System (Bio-Rad, California, United States). Lane ss: single DNA with 3′-fluorescein (S43; shown in Table 1). Top of the arrow: annealed double-stranded DNA; bottom of the arrow: unannealed free ssDNA(S43). The results of **(C,D)** were visualized by a ChemiDoc MP Imaging System (Bio-Rad, California, United States).

Both CoV-2 N and CoV-2 Nsp13 exhibit strong binding activity to ssDNA, and this activity can often promote the annealing of two complementary ssDNA molecules (Figures 7A,B). The substrates used in Figures 2A,B were dsDNA molecules, which had been successfully annealed before the reaction, and the unwinding process was observed. To gain insights on the annealing properties of CoV-2 N and CoV-2 Nsp13, the nucleic acid substrates used in Figures 7C,D were two partially complementary paired ssDNAs (S43 and S21) that did not exhibit annealing. The experiments were performed in buffer A and buffer B using an *in vitro* gel assay system. Typical helicase-dependent unwinding requires ATP hydrolysis, whereas DNA annealing usually does not require ATP. This is one of the reasons why no ATP was included in the reaction. Another important factor is that annealing and unwinding are performed simultaneously after the addition of ATP; thus, the annealing activity cannot be observed. Gradually increasing concentrations of CoV-2 N and CoV-2 Nsp13 were added in the reaction solution containing two partially complementary ssDNAs (S43 and S21). The optimal annealing concentration of CoV-2 Nsp13 was markedly different from that of CoV-2 N (Figures 7C,D). The annealing ratio of CoV-2 N at the optimum concentration reached 59.4%, whereas that of CoV-2 Nsp13 was only 6.5%. The annealing activity of CoV-2 N was almost 10 times that of CoV-2 Nsp13 (Supplementary Figure 7). Our results demonstrated that CoV-2 N does not need to hydrolyze ATP during dsDNA unwinding. Thus, CoV-2 N exhibits the two opposite activities of unwinding and annealing at different concentrations in the same reaction buffer. However, considering that the concentration of CoV-2 N required for annealing is much lower than that required for unwinding, the main function of CoV-2 N is likely annealing.

## Discussion

CoV-2 N is the only protein that binds to genomic RNA in the nucleocapsid. It is closely related to gene replication and cell signaling pathway regulation. The N protein is one of the most conserved proteins of coronaviruses (Zhou et al., 2020; Thye et al., 2021). CoV-2 Nsp13 significantly contributes to viral replication and exhibits the highest sequence conservation among coronaviruses, highlighting its importance in viral viability (Adedeji et al., 2012; Yu et al., 2012). This enzyme represents a promising target for the development of drugs against coronaviruses (Adedeji and Sarafianos, 2014). Therefore, it is necessary to systematically analyze the biochemical characteristics of CoV-2 N and CoV-2 Nsp13 *in vitro*. Based on the published sequences, we successfully expressed and purified two proteins, corroborating the findings of previous reports (Zeng et al., 2020; Newman et al., 2021).

**Figure 8:**
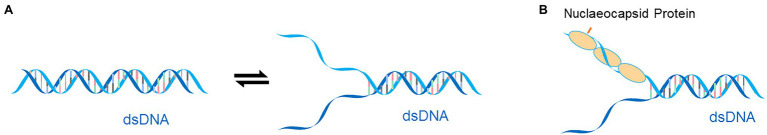
**(A)** The dynamic self-dissociation of dsDNA. **(B)** The dsDNA melting model of CoV-2 N-protein.

The results of this and previous studies indicate that CoV-2 Nsp13 exhibits unwinding activity (Mickolajczyk et al., 2021). However, no specific reports indicate that CoV-2 N or other coronavirus nucleocapsid proteins exhibit unwinding activity. The two proteins exhibited little similarity in their primary sequences. CoV-2 Nsp13 contains structurally conserved domains of typical helicases, such as ATP-binding and ATP-hydrolysis sites, whereas CoV-2 N does not contain any domain with predicted unwinding function (Supplementary Figures 1 and 2). This suggests that the N protein, which resembles a single-strand binding protein, opens duplexes via a mechanism that is different to typical helicases (Figure 8; McBride et al., 2014; Kang et al., 2020; Zeng et al., 2020).

An *in vitro* gel assay system was established for the first time to determine the effect of the unwinding activity of CoV-2 N *in vitro*. Unlike CoV-2 Nsp13, we found that CoV-2 N was not a helicase. CoV-2 N does not require Mg^2+^, DTT, or NTP to unwind dsDNA. CoV-2 N unwound dsDNA in the presence of 100–300 mM NaCl. Conversely, CoV-2 Nsp13 exhibited superior unwinding activity at low NaCl concentrations. Our binding experiments revealed that concentrations of NaCl of >400 mM inhibited CoV-2 N binding to ssDNA (the result is not show). This may explain why the unwinding ability of CoV-2 N becomes weaker at high salt concentrations (Figure 3A). Low salt concentration is known to affect the binding of N protein to ssDNA, possibly due to charge effects. It is worth noting that the unwinding activity of CoV-2 N protein does not require ATP and Mg^2+^, two essential cofactors for helicases. Therefore, the unwinding properties of CoV-2 N are probably similar to those of RPA owing to its helix-destabilizing activity, rather than helicase activity. However, CoV-2 N does not unwind dsDNA in the same way as typical SSB proteins, which require a specific temperature (Wang et al., 2004; Safa et al., 2016). Our results indicated that CoV-2 N completely unwound dsDNA at 25°C and 30°C. RPA is extremely heat-labile and very sensitive to freezing and thawing. Compared with CoV-2 N and CoV-2 Nsp13, the effect of temperature and time was somewhat consistent. Both proteins can unwind dsDNA, but they require very different unwinding environments. CoV-2 Nsp13, the only protein with an unwinding domain in the SARS-CoV-2 genome, is very important for viral replication and recombination. The properties of DNA and RNA are similar, and CoV-2 N may also act on viral RNA in a similar manner. CoV-2 N is more likely a supplement for CoV-2 Nsp13 under the conditions of stress, such as a lack of ATP. However, the mode of their action is very different, and it is possible that the two function independently at different stages of viral replication.

Our findings demonstrated that the concentration of N protein required for dsDNA unwinding was much higher (4 μM) than that of Nsp13 (0.3 μM) (Figures 2A,B). This is clearly applicable for CoV-2 N, which is a multifunctional protein and not a typical helicase, and may mean that multifunctional proteins should be present at higher concentrations in vivo to perform this function. For example, telomerase, which is essentially a polymerase but not a typical polymerase, is irreplaceable in all organisms. Compared with typical reverse transcriptases and polymerases, the polymerization activity of telomerase is only 1/10,000. In addition, nucleocapsid protein is the most abundant viral protein, with concentrations of up to 10^8^ molecules in an infected cell (Tugaeva et al., 2021). Considering that the volume of human lung cells is approximately 170 μm^3^, the concentration of nucleocapsid protein can reach nearly 1 mM, which is much higher than the concentrations used in our unwinding experiments.

Although CoV-2 N and CoV-2 Nsp13 exhibit the same unwinding polarity, they show differences in unwinding substrates. The present study demonstrates that CoV-2 N has strict requirements for tail-chain length, only unwinding substrates with a 5′overhang of 16 nucleotides. This suggests that similar to RPA, the unwinding activity of CoV-2 N depends on ssDNA length. CoV-2 Nsp13 showed properties similar to typical helicases—its unwinding activity was exhibited only when the single-stranded bubble structure was at least 12 nucleotides in length and activity increased with increased tail length (Figure 6B). However, CoV-2 N could not open substrates with bubble structures of any length (Figure 6A).

Our findings of the difference in CoV-2 N and CoV-2 Nsp13 annealing activities at equivalent and increasing concentrations indicate that the annealing activity of CoV-2 N is active at low concentrations, whereas the unwinding activity predominates at high concentrations. This may be because high amounts of CoV-2 N can saturate ssDNA and eliminate the secondary structure, so that the ssDNA is in a simple state that is conducive to binding by other proteins. The proteins bound to the nucleic acid protect the ssDNA. It is also possible that CoV-2 N regulates the transition between annealing and unwinding based on its concentration. Thus, annealing is likely to be the primary function of CoV-2 N.

The role of the unwinding function of CoV-2 N in viruses and host cells remains unclear, and further studies are needed. A reasonable hypothesis regarding its function may relate to the different subcellular localization of the N protein in host cells. The N protein of coronavirus may be localized in the cytoplasm and nucleus of host cells (Rowland et al., 1999; Hiscox et al., 2001; Wurm et al., 2001; Timani et al., 2005). In the cytoplasm, this protein participates in viral genome replication, transcription, and packaging through its RNA-binding and -annealing activities (McBride et al., 2014). In the nucleus, this protein delays the cell cycle in the G2/M phase by relocalizing into the nucleus through its nuclear localization sequence (Wurm et al., 2001). Early studies on SARS-CoV N protein showed the protein also exhibits high nucleolar localization in the G2/M phase in SARS-CoV–infected cells (Cawood et al., 2007). In contrast, recent studies on SARS-CoV-2 transfection revealed that the N protein was also apparent in the G1/S phase of the cell cycle (Gao et al., 2021). The S phase of the cell cycle represents the DNA replication phase. Thus, the ssDNA binding activity and dsDNA unwinding activity of the SARS-CoV-2 N protein observed in this study indicate that this protein binds to the host’s genomic ssDNA during replication and affects host cell replication. This creates favorable conditions for the packaging of SARS-CoV-2.

## Data Availability Statement

The original contributions presented in the study are included in the article/Supplementary Material; further inquiries can be directed to the corresponding authors.

## Author Contributions

BZ and YL are responsible for the experiments design. BZ, YX, and ZL performed experiments. BZ, DL, and QZ compiled figures. BZ, KL, and YL wrote and edited the manuscript. BZ, YX, ZL, DL, JT, QZ, HT, JY, XZ, SQ, and YL analyzed and interpreted the data. All authors contributed to the article and approved the submitted version.

## Funding

This work was supported by the Guizhou Province Science and Technology Plan Foundation [grant number (2020)4Y204], the Science and Technology Top-notch Talents Foundation of General Colleges and Universities in Guizhou Province QIAN JIAO HE KY ZI [grant number (2021)035], the Science and Technology Fund Project of Guizhou Provincial Health Commission [grant number (2020)170], and the National Natural Science Foundation of China (31860315).

## Conflict of Interest

The authors declare that the research was conducted in the absence of any commercial or financial relationships that could be construed as a potential conflict of interest.

## Publisher’s Note

All claims expressed in this article are solely those of the authors and do not necessarily represent those of their affiliated organizations, or those of the publisher, the editors and the reviewers. Any product that may be evaluated in this article, or claim that may be made by its manufacturer, is not guaranteed or endorsed by the publisher.
