# Supplementary material for: SARS-CoV-2 Nucleocapsid Protein Has DNA-Melting and Strand-Annealing Activities With Different Properties From SARS-CoV-2 Nsp13
Source: Front Microbiol. 2022 Jul 22;13:851202. doi: 10.3389/fmicb.2022.851202 (PMC9354549; doi:10.3389/fmicb.2022.851202)
Supplement: Supplementary file 1 [file Data_Sheet_1.zip › Supplement -to typesetter1/Supplement 1/Supplement.1-Fig Lenged.docx]

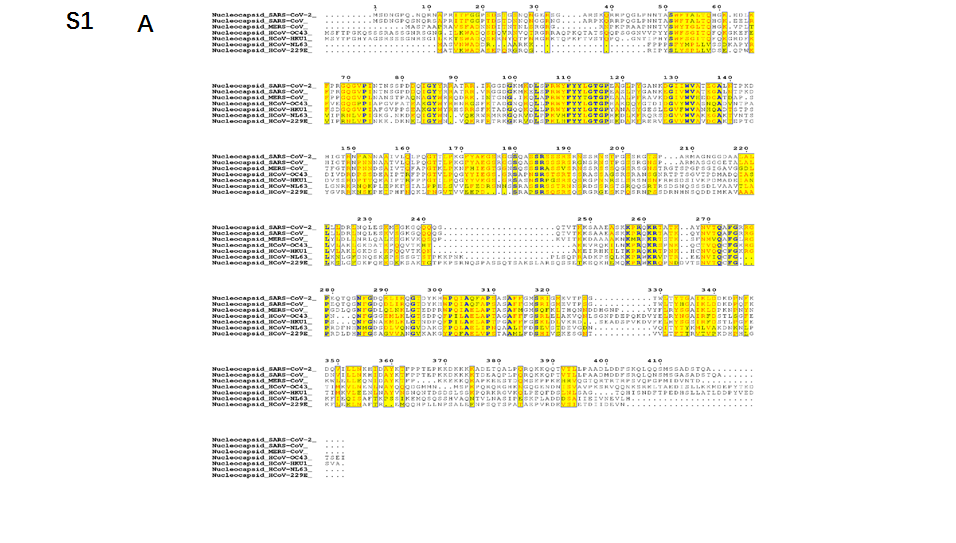


Supplement.1 (A) Sequence comparison of seven HCoVs Nucleocapsid protein: Nucleocapsid-SARS-CoV-2 (UniProtKB: P0DTC9),

Nucleocapsid-SARS-CoV (UniProtKB: P59595),

Nucleocapsid-MERS-CoV (UniProtKB: K9N4V7),

Nucleocapsid-HCoV-OC43 (UniProtKB: P33469),

Nucleocapsid-HCoV-NL63 (UniProtKB: Q6Q1R8),

Nucleocapsid-HCoV-HKU1 (UniProtKB: Q5MQC6),

Nucleocapsid-HCoV-229E (UniProtKB: P15130).


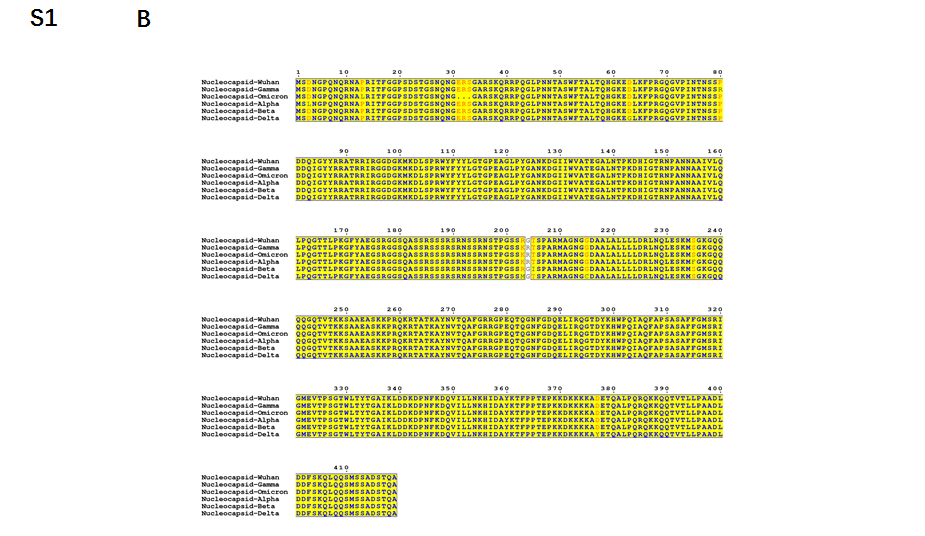


Supplement.1 (B) Multiple sequence alignment of VOC (variants of concern) of the SARS-CoV-2 Nucleocapsid proteins:

Nucleocapsid-Alpha B.1.1.7 (GenBank: QWE88928.1),

Nucleocapsid-Beta B.1.351 (GenBank: QRN78355.1),

Nucleocapsid-Gamma P.1 (B.1.1.28.1)(GenBank: QVE55297.1),

Nucleocapsid-Delta B.1.617.2 (GenBank: QWK65237.1),

Nucleocapsid-Omicron B.1.1.529 ( GenBank: UFO69287.1).


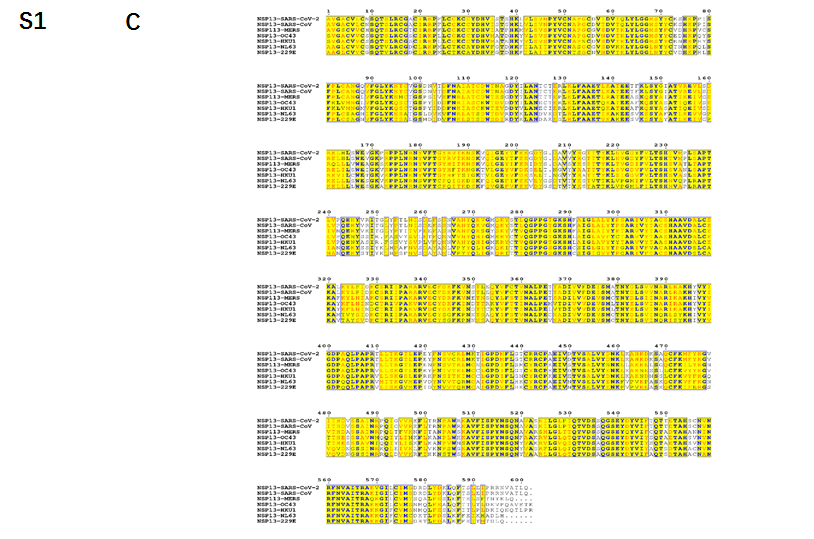


Supplement.1 (C) Sequence comparison of seven HCoVs NSP13 protein:

NSP13-SARS-CoV-2 (UniProtKB: P0DTD1),

NSP13-SARS-CoV (UniProtKB: P0C6X7),

NSP13-MERS-CoV (UniProtKB: K9N7C7),

NSP13-HCoV-OC43 (UniProtKB: P0C6X6),

NSP13-HCoV-NL63 (UniProtKB: P0C6X5),

NSP13-HCoV-HKU1 (UniProtKB: P0C6X2),

NSP13-HCoV-229E (UniProtKB: P0CX61).


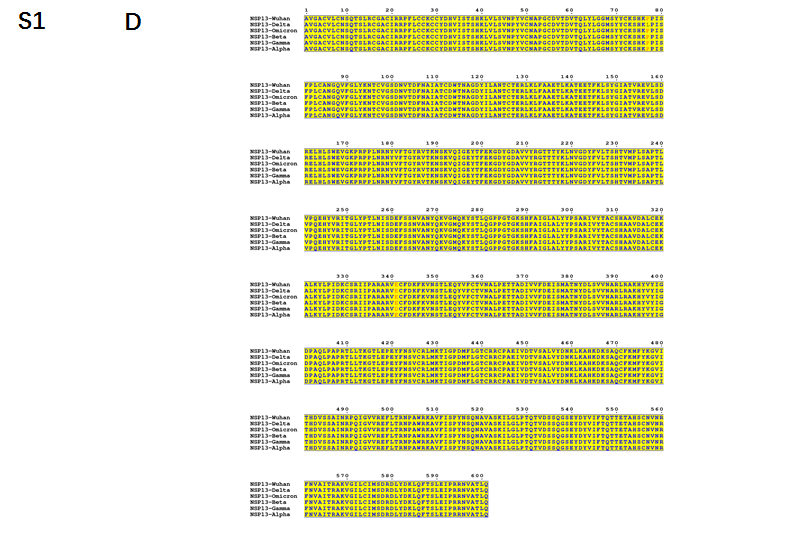


Supplement.1 (D) Multiple sequence alignment of VOC (variants of concern) of the SARS-CoV-2 NSP13 proteins:

NSP13-Alpha B.1.1.7 (GenBank: MZ344997.1),

NSP13-Beta B.1.351 (GenBank: MW598419.1),

NSP13-Gamma P.1(B.1.1.28.1)(GenBank:  MZ169911.1),

NSP13-Delta B.1.617.2(GenBank: MZ359841.1),

NSP13-Omicron B.1.1.529 ( GenBank: OL672836.1).
