# Supplementary material for: SARS-CoV-2 Nucleocapsid Protein Has DNA-Melting and Strand-Annealing Activities With Different Properties From SARS-CoV-2 Nsp13
Source: Front Microbiol. 2022 Jul 22;13:851202. doi: 10.3389/fmicb.2022.851202 (PMC9354549; doi:10.3389/fmicb.2022.851202)
Supplement: Supplementary file 1 [file Data_Sheet_1.zip › Supplement -to typesetter1/Supplement 2/Supplement.2-Fig Lenged.docx]

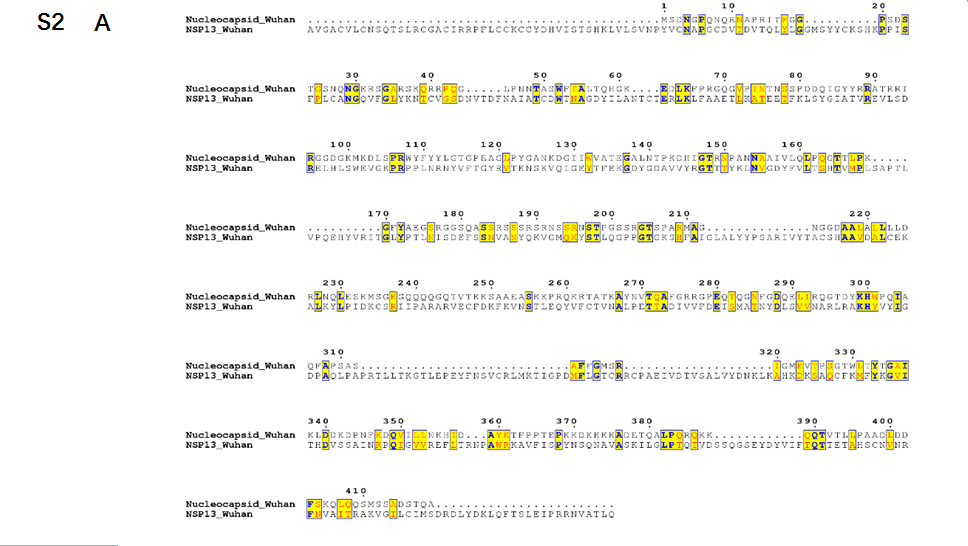


Supplement 2 (A) Sequence alignment of SARS-CoV-2 N protein and NSP13: Uncleocapsid-Wuhan (UniProtKB: P0DTC9), NSP13-Wuhan(UniProtKB:P0DTD1).


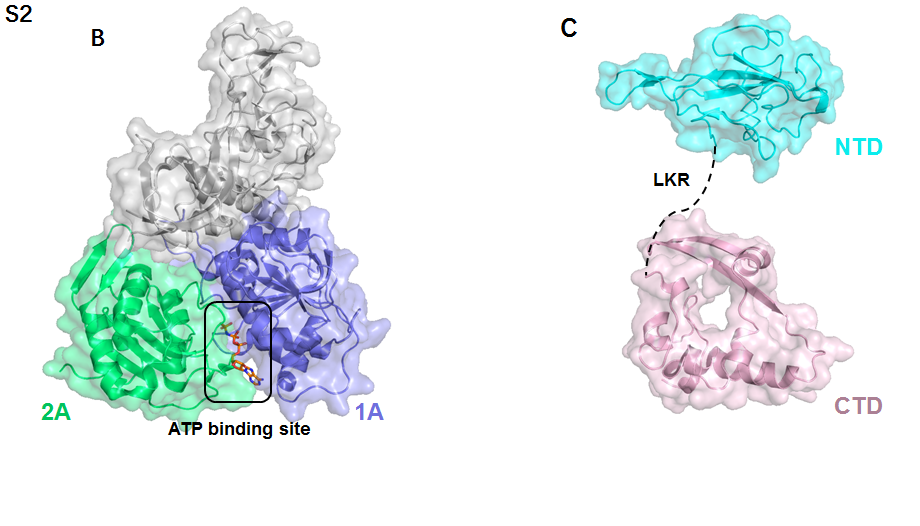


Supplement 2 (B) X-ray structures of SARS-CoV-2 NSP13 helicase (PDB:7NN0). The RecA-like domains are colored in green and blue, respectively. The ATP analogues (AMP-PNP) located in the cleft of RecA domains is colored in orange. (C) The putative structures of SARS-CoV-2 Nucleocapsid protein. The N-Terminal-Domain (NTD) (PDB:7CDZ) and C-Terminal-Domain (CTD) (PDB:6WJI) ARE colored in cyan and pink, respectively. The unsolved disordered central linker region (LKR) is presented in dotted line.
