# Supplementary material for: SARS-CoV-2 Nucleocapsid Protein Has DNA-Melting and Strand-Annealing Activities With Different Properties From SARS-CoV-2 Nsp13
Source: Front Microbiol. 2022 Jul 22;13:851202. doi: 10.3389/fmicb.2022.851202 (PMC9354549; doi:10.3389/fmicb.2022.851202)
Supplement: Supplementary file 1 [file Data_Sheet_1.zip › Supplement -to typesetter1/Supplement 3/Supplement.3-Fig Lenged.docx]

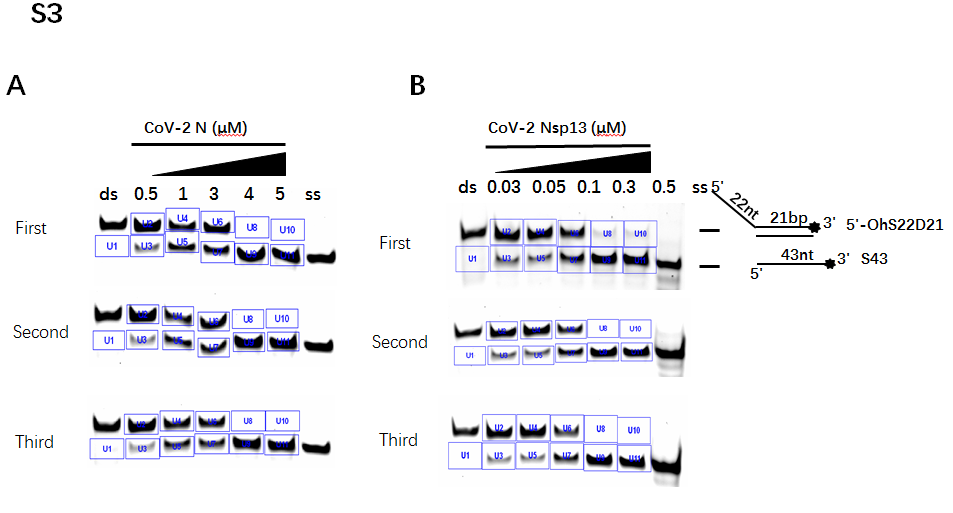


Supplement 3. (A and B) DNA was quantitated as shown above by using the Image Lab software (Bio-Rad) to get the adjusted volume, and ues it to calculate the fraction using the following formula：$\%unwinding=100\times\frac{P}{S+P}$, P is the product and S is the substrate. Take OhS22D21 as an example： $\% unwinding=100\times\frac{U3－U1}{U2＋U3－U1}$.where U3 is the product, U2 is the substrate, U1 is the spontaneously unwind product, U3-U1 is the CoV-2 N / CoV-2 Nsp 13 unwinding product.

| **The original data of the unwinding ratio** | | | | | |
| --- | --- | --- | --- | --- | --- |
| **Unwinding (%)** | **First** | **Second** | **Third** | **Average** | **Stdev** |
| **CoV-2 N concentration（3uM）** | 0.402501621 | 0.424220284 | 0.429755176 | 0.418826 | 0.014405 |
| **CoV-2 Nsp13 concentration(0.3uM)** | 0.947556122 | 0.960780947 | 0.956808516 | 0.955049 | 0.006786 |
|  |  |  |  |  |  |
|  |  |  |  |  |  |


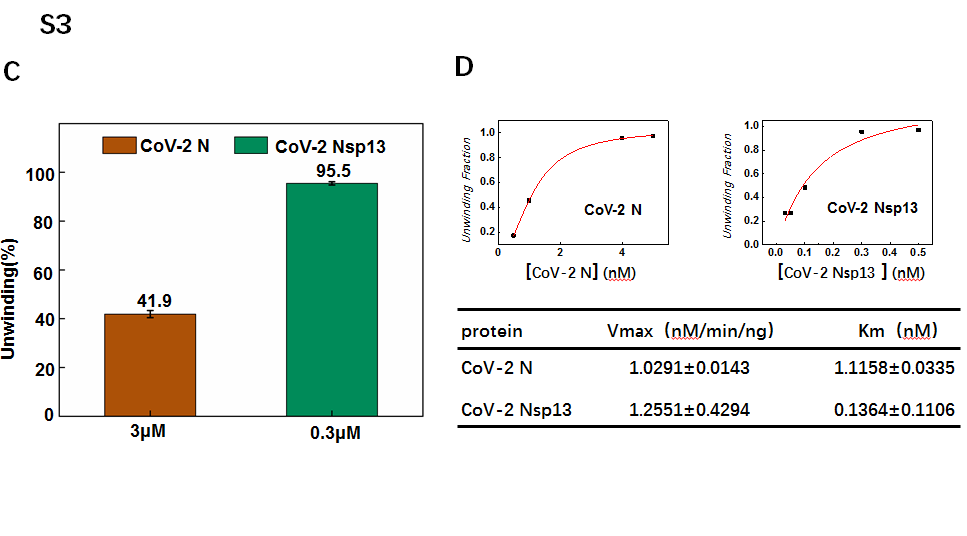


Supplement 3. (C) Comparison of CoV-2 N 3uM and CoV-2 Nsp13 0.3uM unwinding fraction; (D) Kinetics of CoV-2 N and CoV-2 Nsp13 helicase activity. Unwinding assay reactions for CoV-2 N and CoV-2 Nsp13 were performed using the substrate (OhS22D21) of different concentrations in (Figure 2A and B) in a standard reaction buffer A / buffer B. Using Image software the amount of dsDNA and unwound ssDNA was quantified from the autoradiogram and used for the Km and Vmax calculations.
