# Supplementary material for: SARS-CoV-2 Nucleocapsid Protein Has DNA-Melting and Strand-Annealing Activities With Different Properties From SARS-CoV-2 Nsp13
Source: Front Microbiol. 2022 Jul 22;13:851202. doi: 10.3389/fmicb.2022.851202 (PMC9354549; doi:10.3389/fmicb.2022.851202)
Supplement: Supplementary file 1 [file Data_Sheet_1.zip › Supplement -to typesetter1/Supplement 4/Supplement.4-Fig Lenged.docx]

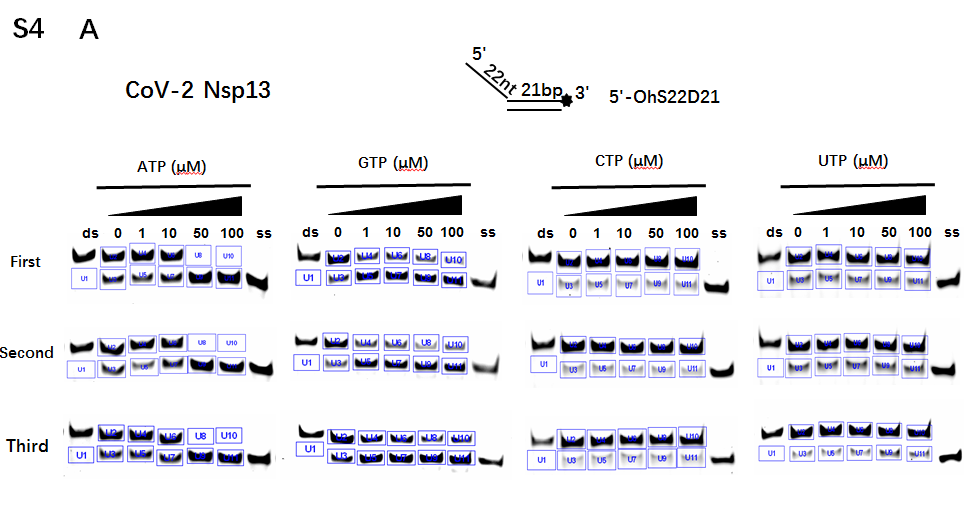


Supplement 4. (A) DNA was quantitated as shown above by using the Image Lab software (Bio-Rad) to get the adjusted volume, and use it to calculate the fraction using the following formula：$\%unwinding=100\times\frac{P}{S+P}$, P is the product and S is the substrate. Take OhS22D21 as an example： $\% unwinding=100\times\frac{U3－U1}{U2＋U3－U1}$.where U3 is the product, U2 is the substrate, U1 is the spontaneously unwind product, U3-U1 is the CoV-2 Nsp 13 unwinding product.

| **The original data of the unwinding ratio** | | | | | |
| --- | --- | --- | --- | --- | --- |
| **Unwinding (%)** | **First** | **Second** | **Third** | **Average** | **Stdev** |
| **ATP concentration(10uM)** | 0.38671295 | 0.43904446 | 0.368599174 | 0.398118861 | 0.036581 |
| **GTP concentration(10uM)** | 0.6445686 | 0.630427592 | 0.637024569 | 0.637340253 | 0.007076 |
| **CTP concentration(10uM)** | 0.152188634 | 0.151984346 | 0.159902942 | 0.154691974 | 0.004514 |
| **UTP concentration(10uM)** | 0.257014296 | 0.285308573 | 0.229000063 | 0.257107644 | 0.028154 |
|  |  |  |  |  |  |
|  |  |  |  |  |  |
| **The original data of the unwinding ratio** | | | | | |
| **Unwinding (%)** | **First** | **Second** | **Third** | **Average** | **Stdev** |
| **ATP concentration(50uM)** | 0.99270656 | 0.990006455 | 0.977972073 | 0.98689503 | 0.007845 |
| **GTP concentration(50uM)** | 0.696627853 | 0.728952611 | 0.735867515 | 0.72048266 | 0.020946 |
| **CTP concentration(50uM)** | 0.162860117 | 0.15169127 | 0.158441313 | 0.157664233 | 0.005625 |
| **UTP concentration(50uM)** | 0.257687883 | 0.268218017 | 0.293403192 | 0.273103031 | 0.018352 |
|  |  |  |  |  |  |


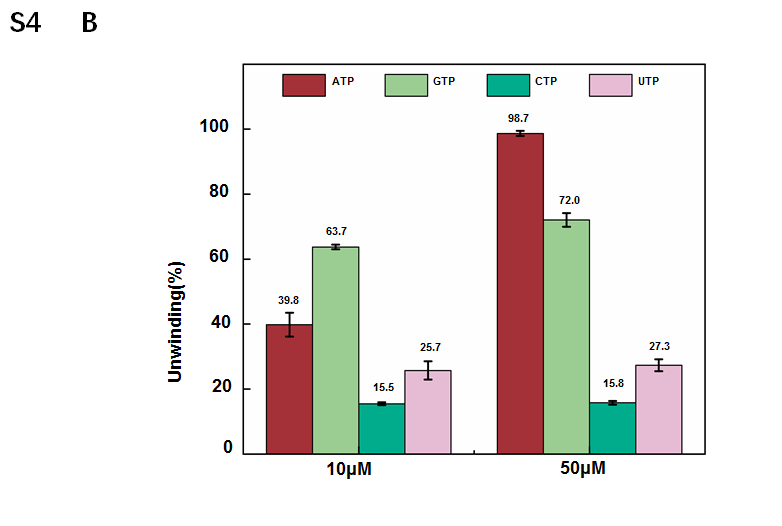


Supplement 4 (B) Effects of different Nucleotide triphosphate (NTP) on the unwinding ability of CoV-2 Nsp13.
