# Supplementary material for: SARS-CoV-2 Nucleocapsid Protein Has DNA-Melting and Strand-Annealing Activities With Different Properties From SARS-CoV-2 Nsp13
Source: Front Microbiol. 2022 Jul 22;13:851202. doi: 10.3389/fmicb.2022.851202 (PMC9354549; doi:10.3389/fmicb.2022.851202)
Supplement: Supplementary file 1 [file Data_Sheet_1.zip › Supplement -to typesetter1/Supplement 5/Supplement.5-Fig Lenged.docx]

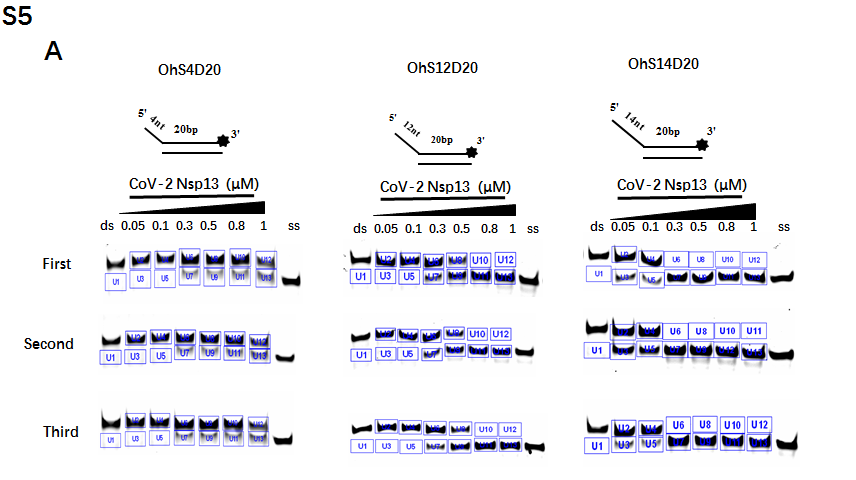


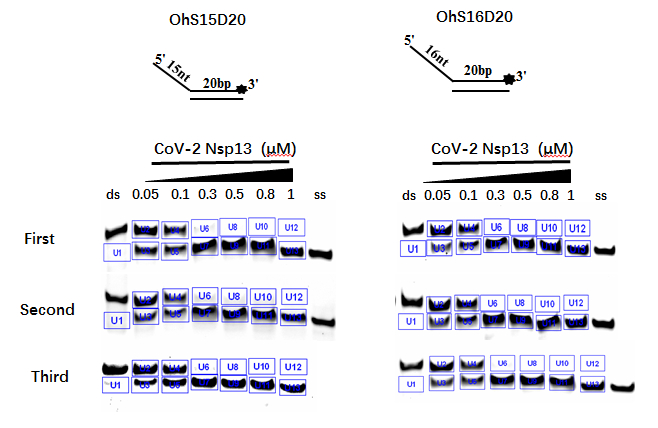


Supplement 5. (A) DNA was quantitated as shown above by using the Image Lab software (Bio-Rad) to get the adjusted volume, and use it to calculate the fraction using the following formula：$\%unwinding=100\times\frac{P}{S+P}$, P is the product and S is the substrate. Take OhS4D20 as an example： $\% unwinding=100\times\frac{U3－U1}{U2＋U3－U1}$.where U3 is the product, U2 is the substrate, U1 is the spontaneously unwind product, U3-U1 is the CoV-2 Nsp 13 unwinding product.

|  |  |  |  |  |  |
| --- | --- | --- | --- | --- | --- |
|  |  |  |  |  |  |
| **The original data of the unwinding ratio** | | | | | |
| **CoV-Nsp13 0.1uM Unwinding (%)** | **First** | **Second** | **Third** | **Average** | **Stdev** |
| **OhS4D20** | 0.030430965 | 0.03674825 | 0.044131677 | 0.03710363 | 0.006857 |
| **OhS12D20** | 0.021377353 | 0.00678031 | 0.001247621 | 0.00980176 | 0.010399 |
| **OhS14D20** | 0.322457518 | 0.326297309 | 0.154376027 | 0.26771028 | 0.098169 |
| **OhS15D20** | 0.456805683 | 0.443494037 | 0.437051327 | 0.44578368 | 0.010074 |
| **OhS16D20** | 0.44962115 | 0.455295241 | 0.467162112 | 0.4573595 | 0.008951 |
|  |  |  |  |  |  |
|  |  |  |  |  |  |
| **The original data of the unwinding ratio** | | | | | |
| **CoV-Nsp13 0.3uM Unwinding (%)** | **First** | **Second** | **Third** | **Average** | **Stdev** |
| **OhS4D20** | 0.220303737 | 0.197695107 | 0.199298183 | 0.20576568 | 0.012616 |
| **OhS12D20** | 0.253535611 | 0.237074268 | 0.251608138 | 0.24740601 | 0.008999 |
| **OhS14D20** | 0.244569687 | 0.236339251 | 0.180885058 | 0.220598 | 0.034638 |
| **OhS15D20** | 0.947981796 | 0.944747004 | 0.880362487 | 0.92436376 | 0.038141 |
| **OhS16D20** | 0.981062498 | 0.980202463 | 0.945467924 | 0.96891096 | 0.020307 |
|  |  |  |  |  |  |
|  |  |  |  |  |  |
|  |  |  |  |  |  |


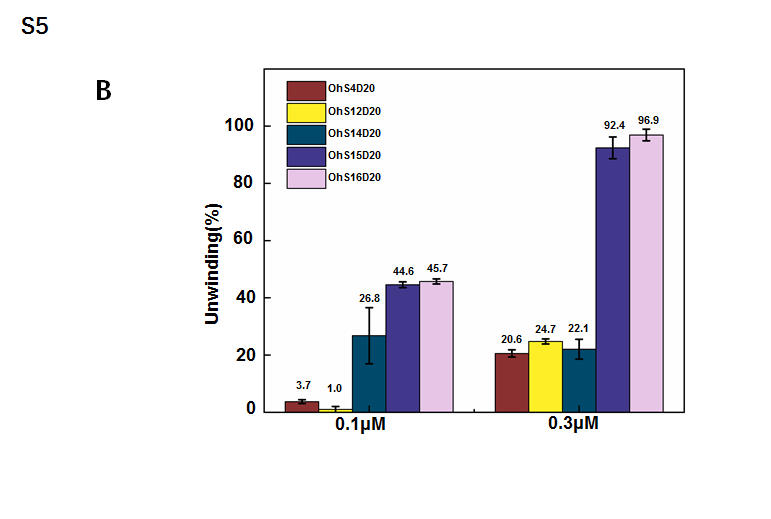


Supplement 5. (B) Unwinding fraction analysis of the various single-stranded overhang length DNA substrates with 0.1μM and 0.3μM concentrations of CoV-2 Nsp13.
