# Supplementary material for: SARS-CoV-2 Nucleocapsid Protein Has DNA-Melting and Strand-Annealing Activities With Different Properties From SARS-CoV-2 Nsp13
Source: Front Microbiol. 2022 Jul 22;13:851202. doi: 10.3389/fmicb.2022.851202 (PMC9354549; doi:10.3389/fmicb.2022.851202)
Supplement: Supplementary file 1 [file Data_Sheet_1.zip › Supplement -to typesetter1/Supplement 6/Supplement.6-Fig Lenged.docx]

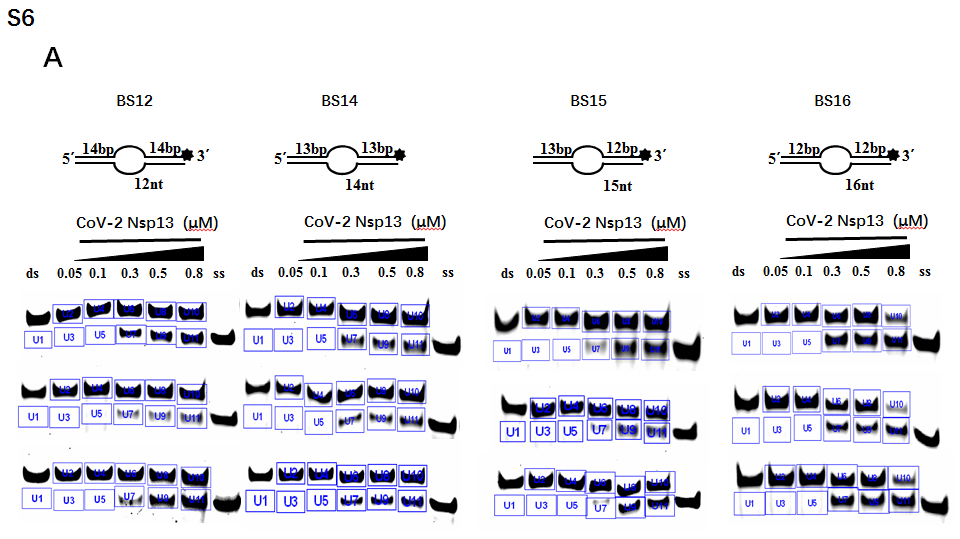


Supplement 6. (A) DNA was quantitated as shown above by using the Image Lab software (Bio-Rad) to get the adjusted volume, and use it to calculate the fraction using the following formula：$\%unwinding=100\times\frac{P}{S+P}$, P is the product and S is the substrate. Take BS12 as an example： $\% unwinding=100\times\frac{U3－U1}{U2＋U3－U1}$.where U3 is the product, U2 is the substrate, U1 is the spontaneously unwind product, U3-U1 is the CoV-2 Nsp 13 unwinding product.

|  |  |  |  |  |  |
| --- | --- | --- | --- | --- | --- |
| **The original data of the unwinding ratio** | | | | | |
| **CoV-Nsp13 0.3uM Unwinding (%)** | **First** | **Second** | **Third** | **Average** | **Stdev** |
| **BS12** | 0.126832694 | 0.134017527 | 0.140201369 | 0.133684 | 0.00669058 |
| **BS14** | 0.161911007 | 0.149439692 | 0.146580199 | 0.152644 | 0.008152137 |
| **BS15** | 0.159368919 | 0.155391492 | 0.192980625 | 0.169247 | 0.020649898 |
| **BS16** | 0.439177328 | 0.387283992 | 0.435186501 | 0.420549 | 0.028877602 |
|  |  |  |  |  |  |
|  |  |  |  |  |  |
| **The original data of the unwinding ratio** | | | | | |
| **CoV-Nsp13 0.8uM Unwinding (%)** | **First** | **Second** | **Third** | **Average** | **Stdev** |
| **BS12** | 0.217395013 | 0.208194462 | 0.361647681 | 0.262412 | 0.086063322 |
| **BS14** | 0.200339503 | 0.225805334 | 0.188532215 | 0.204892 | 0.019049086 |
| **BS15** | 0.382965885 | 0.365429356 | 0.422691832 | 0.390362 | 0.02933903 |
| **BS16** | 0.677924988 | 0.641021547 | 0.641237557 | 0.653395 | 0.021244129 |


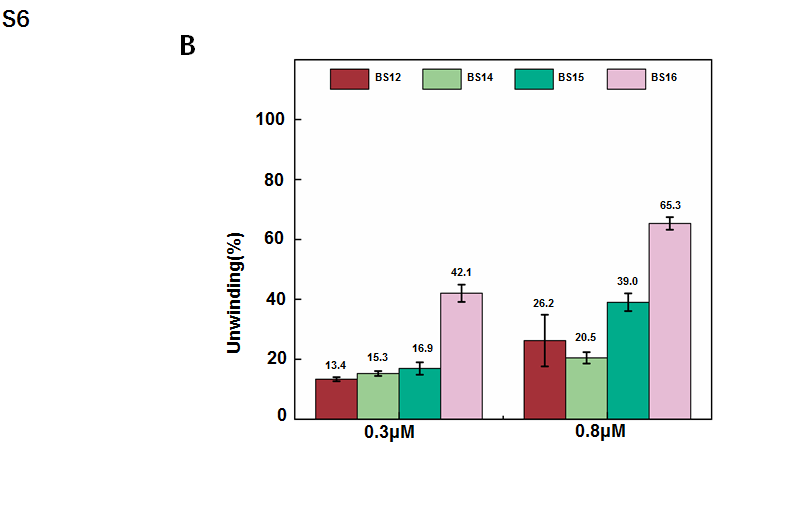


Supplement 6. (B) Unwinding fraction analysis of the dsDNA bubble structure with 0.3μM and 0.8μM concentrations of CoV-2 Nsp13.
