# Supplementary material for: SARS-CoV-2 Nucleocapsid Protein Has DNA-Melting and Strand-Annealing Activities With Different Properties From SARS-CoV-2 Nsp13
Source: Front Microbiol. 2022 Jul 22;13:851202. doi: 10.3389/fmicb.2022.851202 (PMC9354549; doi:10.3389/fmicb.2022.851202)
Supplement: Supplementary file 1 [file Data_Sheet_1.zip › Supplement -to typesetter1/Supplement 7/Supplement.7-Fig Lenged.docx]

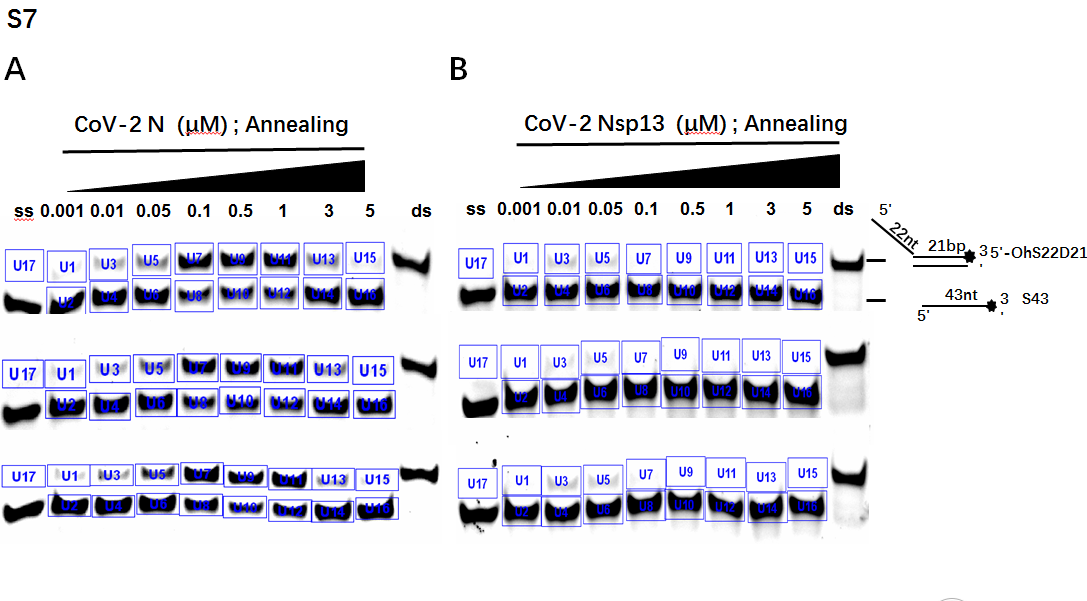


Supplement 7 (A and B) DNA was quantitated as shown above by using the Image Lab software (Bio-Rad) to get the adjusted volume, and use it to calculate the fraction using the following formula：$\%Annealing=100\times\frac{P}{S+P}$, P is the product and S is the substrate. Take OhS22D21 as an example： $\% Annealing=100\times\frac{U1－U17}{U1＋U2－U17}$.where U1 is the product dsDNA-OhS22D21, U2 is the substrate S43, U17 is the spontaneously S43 product, U1-U17 is the annealing product.

|  | |  |  | |  |  |  | |  |  |  |
| --- | --- | --- | --- | --- | --- | --- | --- | --- | --- | --- | --- |
| **The original data of the annealing ratio** | | | | | | | | | | | |
| CoV-2 N(uM) | First | | | Second | | | | Third | | AVERAGE | STDEV |
| 0.5 | 0.612269312 | | | 0.564865655 | | | | 0.597054774 | | 0.59139658 | 0.019761715 |
|  |  | | |  | | | |  | |  |  |
| **The original data of the annealing ratio** | | | | | | | | | | | |
| CoV-Nsp 13 | First | | | Second | | | | Third | | AVERAGE | STDEV |
| 0.05 | 0.077943973 | | | 0.02779504 | | | | 0.087782252 | | 0.06450709 | 0.026268216 |
|  |  | | |  | | | |  | |  |  |


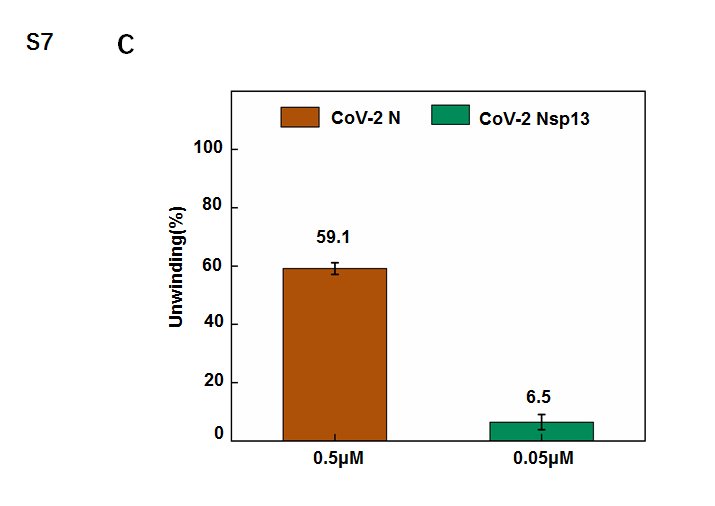


Supplement 7 (C) The comparison of annealing activity of CoV-2 N at 0.5μM and CoV-2 Nsp13 at 0.05μM.
